# Assessment of Crack Resistance and Influencing Factors of Ultra-Thin Asphalt Overlay Mixtures Using SCB Testing

**DOI:** 10.3390/ma19173742

**Published:** 2026-09-03

**Authors:** Yaofang Zhang, Chongsheng Xin, Jiyuan Tian, Fengli Lv, Qing Wang, Baodong Xing, Chuanyi Zhuang

**Affiliations:** 1 School of Transportation and Civil Engineering, Shandong Jiaotong University, Jinan 250357, China; 2 Shandong Key Laboratory of Technologies and Systems for Intelligent Construction Equipment, Shandong Jiaotong University, Jinan 250357, China; 3 Shandong Kingyue Transportation Development Group Co., Ltd., Jinan 250101, China; 4 School of Civil Engineering, Yancheng Institute of Technology, Yancheng 224051, China; gwyxbd@163.com

**Keywords:** ultra-thin overlay, SCB, crack resistance, regression model, key influencing factors

## Abstract

Asphalt ultra-thin overlays have emerged as an efficient and cost-effective pavement preservation technology. However, because of their reduced thickness, their service life is often limited by cracking. Based on semi-circular bending (SCB) test data, this study developed three types of regression models: multiple linear regression (MLR), random forest regression (RFR), and artificial neural network (ANN) regression. The multifactor experimental matrix covered three test temperatures, three nominal maximum aggregate sizes, two binder types, three aging states, and four long-term aging durations. The results show that fracture energy (G_f_), flexibility index (FI), and cracking resistance index (CRI) respond differently to individual factors. G_f_ and FI decreased as temperature increased, whereas CRI increased, indicating that CRI may be misleading at intermediate temperatures and should be interpreted cautiously. The aggregate size exhibits a non-monotonic relationship with the three indicators, reaching its maximum at a size of 8. Short-term aging increased the G_f_, and the gain was more pronounced at higher aging temperature, reaching a maximum increase of up to 1.54 times. However, it also accelerated the subsequent G_f_ reduction during long-term aging, after the decline rate decreased. Higher short-term aging temperatures caused more pronounced deterioration in FI and CRI. Meanwhile, both indices gradually decreased with increasing long-term aging duration, and short-term aging further intensified this reduction. In model comparisons, ANN showed the most stable generalization. The factorwise trends were verified using both the Beta values and SHAP values, demonstrating the feasibility of the models for qualitative discrimination and trend interpretation. The integrated importance ranking identified long-term aging duration and temperature as the dominant factors. External validation further confirmed ANN as the most reliable model for predicting crack resistance evolution. These findings support crack-resistance prediction and provide guidance for optimizing the design of ultra-thin overlay asphalt mixtures.

## 1. Introduction

Under environmental exposure and traffic loading, asphalt pavement skid resistance may deteriorate significantly. In some urban expressway sections, insufficient skid resistance can compromise driving safety and increase tire–pavement noise, even when other pavement performance indicators remain acceptable. Ultra-thin functional overlay is a thin asphalt surface layer with a typical thickness of 15–25 mm, generally not exceeding 25 mm. Due to its limited thickness, it has little influence on pavement elevation, drainage, and clearance and is mainly used as a functional maintenance layer to improve skid resistance, reduce noise, and delay surface deterioration [1]. Ultra-thin overlays can improve skid resistance, noise reduction, and waterproofing while maintaining pavement serviceability. SMA-type ultra-thin overlays are particularly suitable because their stone-on-stone skeleton enhances rutting resistance, while the binder-rich mortar improves raveling resistance and durability [2]. However, field performance can still be limited by cracking. Cracks weaken interlayer bonding and structural continuity, allow water ingress, and accelerate damage propagation, thereby shortening the effective maintenance period [3].

Cracking is one of the most serious forms of asphalt pavement deterioration. It directly reduces structural integrity and shortens service life [4]. Increasing attention has been paid to the effects of material composition, mixture design, and service conditions on the crack resistance of asphalt mixtures [5]. The effects of binder type, aggregate type, and gradation on the cracking performance of asphalt mixtures have been systematically investigated [6]. Aggregate and binder types jointly affect cracking performance and mixture durability. High-viscosity modified binders show good applicability in ultra-thin wearing courses by improving rutting resistance, low-temperature cracking resistance, and moisture stability [7]. Basalt fibers can reinforce asphalt mortar and improve binder-aggregate interaction, thereby enhancing rutting resistance, low-temperature cracking resistance, and moisture stability [8]. Thermo-oxidative aging level and test temperature strongly affect asphalt-mixture cracking resistance. Short-term aging may improve crack resistance, whereas long-term aging generally reduces it [9]. In contrast, ultra-thin overlay mixtures are more sensitive to interfacial bonding, temperature, and aging because of their limited thickness, finer aggregate skeleton, and weaker stress redistribution capacity [10]. After crack initiation, cracks can quickly penetrate the thin surface layer and accelerate functional deterioration. However, the governing factors of crack resistance in fine-graded ultra-thin overlays remain insufficiently understood [11]. Few studies have clarified how multi-stage aging interacts with test temperature, NMAS, and binder type to affect cracking response. Therefore, material selection and performance evaluation for ultra-thin overlays still often rely on experience from coarser mixtures, which may not fully explain crack evolution during service.

In terms of performance testing, fracture-mechanics-based mixture tests and indices have been increasingly adopted to better characterize cracking-related performance in both research and balanced mix design practice [12]. Commonly used methods include the semi-circular bending (SCB) test, four-point bending fatigue test, tensile tests, and the overlay test (OT) [13]. The semi-circular bending (SCB) test is widely used to evaluate asphalt-mixture cracking resistance at intermediate temperatures because of its fracture-mechanics basis, low variability, and simple equipment requirements [14]. The SCB test provides cracking-related indices such as fracture energy (G_f_), flexibility index (FI), and cracking resistance index (CRI), which reflect energy dissipation, crack resistance, and post-peak softening behavior with different sensitivities to material, aging, and specimen factors [15]. Cracking-related indices respond differently to mixture composition, material state, fiber type, and test method, so a single index may not fully characterize cracking resistance [16]. Previous studies have shown that FI generally decreases after aging, mainly due to the increase in the post-peak slope (m), indicating that post-peak indices such as FI are sensitive to aging [17]. Although these methods support performance-oriented evaluation, different metrics may give different mixture rankings because they emphasize different parts of the load–displacement response. Therefore, using a single metric to assess factor effects on asphalt mixtures has inherent limitations.

Data-driven analysis is increasingly used to complement experiments by quantifying factor importance and revealing nonlinear interactions that are difficult to identify through one-factor-at-a-time analysis [18]. Random-forest and other machine-learning models have shown strong ability to predict pavement and mixture performance from multiple input variables [19]. SVR, Extra Trees, and GBR models have shown high accuracy in predicting asphalt-mixture fracture load, highlighting the potential of data-driven methods to capture complex effects of design and material factors on cracking performance [20]. RF, KNN, and MARS models have been used to predict cracking resistance based on CTI, while SHAP analysis identified key factors affecting crack resistance and confirmed the value of data-driven performance prediction [21]. Overall, advanced machine-learning methods can integrate mixture design, aging-related variables, and performance indicators into predictive and interpretable frameworks for asphalt-mixture performance evaluation and optimization [22]. However, systematic datasets for ultra-thin overlays that include binder type, gradation, temperature, multi-stage aging, and multiple cracking indicators remain limited, restricting analysis of key factors and their coupled effects.

Although previous studies have shown that asphalt-mixture cracking behavior is affected by interacting factors such as temperature, loading condition, moisture, binder properties, gradation, and aging, most conclusions are based on conventional mixtures or relatively thick pavement layers. For ultra-thin overlays, the finer aggregate skeleton, limited thickness, and reduced stress redistribution capacity may lead to different cracking responses, especially under coupled temperature and aging effects. In addition, nonlinear behavior within specific temperature or aging ranges makes it difficult for one-factor analysis or linear models to fully describe factor interactions. Therefore, this study addresses the lack of an integrated and interpretable framework for evaluating the coupled effects of material, gradation, temperature, and aging variables on the cracking performance of ultra-thin overlay mixtures. It focuses on whether SCB-based indices provide consistent evaluations, how key factors jointly influence cracking responses, and whether interpretable machine-learning models can offer more comprehensive explanations than conventional linear regression. The results provide a data-driven basis for mixture design and cracking performance evaluation of ultra-thin overlays.

## 2. Materials and Mixture Ratio Design 

### 2.1. Raw Materials

To promote asphalt–aggregate adhesion and improve skeleton strength and abrasion resistance, both coarse and fine aggregates were basalt. Limestone mineral filler was produced by grinding limestone; the basic properties of the aggregates and mineral filler are summarized in Table 1. Lignin fiber was incorporated into SMA-8 and SMA-10 to increase asphalt film thickness, improve the dispersion of the asphalt–filler mastic, and enhance mixture cohesiveness, stability, and durability; the fiber properties are provided in Table 2. The modifier used is a high-viscosity and high-elasticity (HVE) modifier sourced from Guolu Gaoke Engineering Technology Institute Co., Ltd., Beijing, China, prepared by colloid-mill shearing styrene–butadiene–styrene (SBS)-modified asphalt binder with 6% high viscosity and high elastic (HVE) modifier. During the preparation of the modified asphalt binder, the modifier was first added to the SBS-modified asphalt and sheared at 6000 rpm for 1 h at 185 °C. Subsequently, the mixture was maintained at 185 °C for another 1 h to allow sufficient swelling of the modifier. Therefore, both the shearing and swelling stages were conducted under controlled temperature conditions of 185 °C. The properties of the HVE modifier and the HVE composite modified asphalt are reported in Table 3 and Table 4, respectively. Compared with the SBS-modified asphalt, the HVE-modified binder showed higher dynamic viscosity, indicating improved cohesion and high-temperature stability. Although excessive viscosity may affect pumping, aggregate coating, mixing, and compaction, the HVE-modified binder was prepared at 185 °C in this study. Uniform aggregate coating and no obvious segregation were observed during laboratory mixing and compaction, suggesting acceptable constructability under proper temperature control.

### 2.2. Gradation Design for Ultra-Thin Surface Course Asphalt Mixture

**Figure 1:**
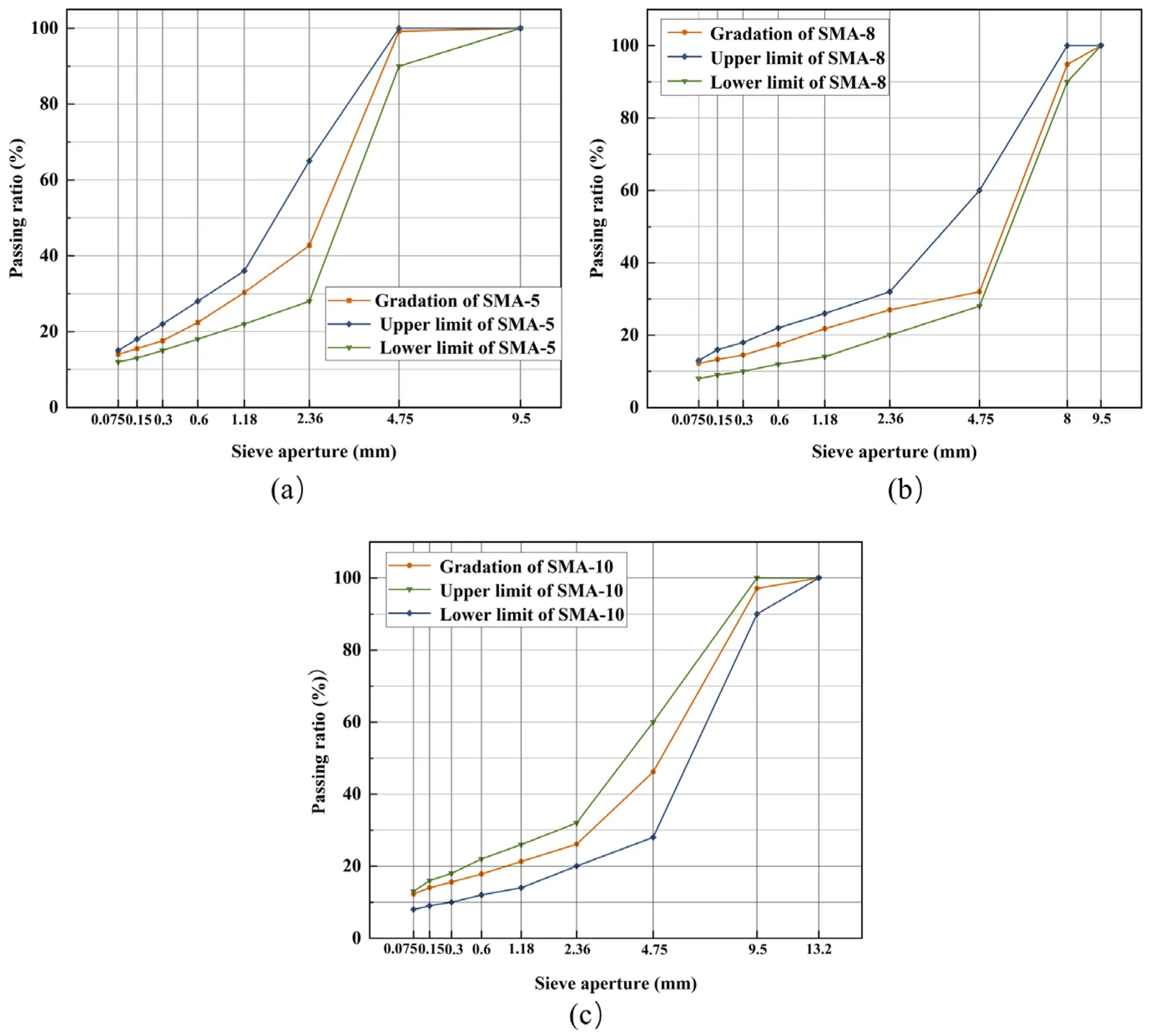
Target gradation distribution and range: (**a**) SMA-5, (**b**) SMA-8, (**c**) SMA-10.

Three SMA mixtures, namely SMA-5, SMA-8, and SMA-10, were selected as candidate asphalt mixtures for ultra-thin overlays. The optimum gradation and binder content of each mixture were determined from Marshall testing by considering volumetric properties, the stone-on-stone criterion (VCA_DRC_ > VCA_mix_), and mixture performance. The selected gradations are presented in Figure 1, and the corresponding optimum binder contents were 6.7%, 6.5%, and 6.2% for SMA-5, SMA-8, and SMA-10, respectively. The Marshall test properties of the specimens are shown in Table 5.

## 3. Experimental Methods

### 3.1. Sample Preparation and Testing Equipment

**Figure 2:**
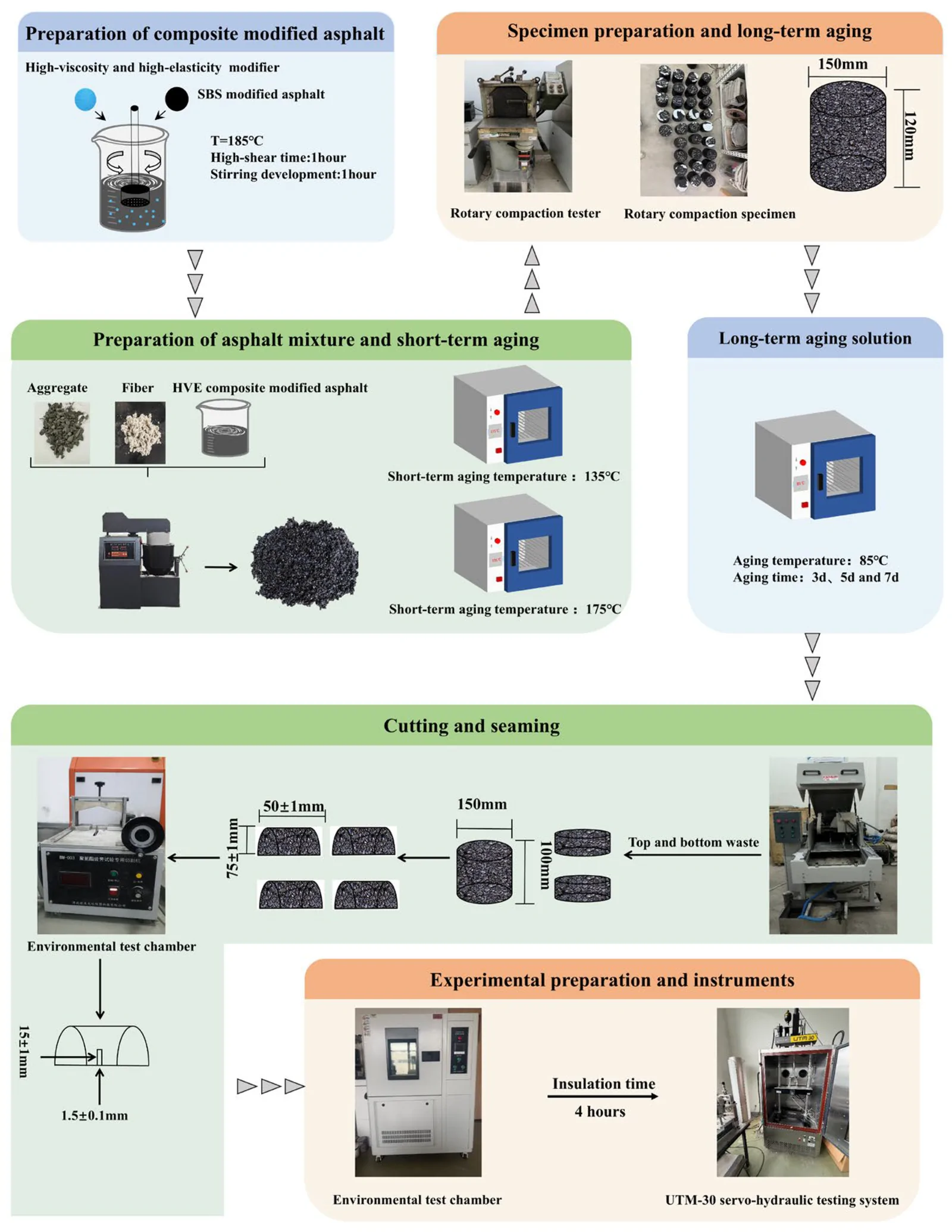
Preparation process and testing equipment for ultra-thin wearing course asphalt mixture specimens.

Specimens were prepared using the Superpave gyratory compactor in accordance with AASHTO T 312 [23]. Compaction was conducted at a gyration angle of 1.25°, an applied pressure of 600 kPa, and a gyration rate of 30 rpm, which simulates field compaction and yields specimens representative of in situ conditions. The gyratory-compacted specimens were initially prepared as cylindrical samples with a diameter of 150 mm and a height of 150 ± 3 mm. Both ends of each specimen were then trimmed to obtain cylindrical specimens with a diameter of 150 mm and a height of 100 mm. Subsequently, the middle section of each trimmed specimen was cut to produce four semi-circular bending (SCB) specimens, each with a diameter of 150 ± 1 mm, a height of 50 ± 1 mm, and a thickness of 75 ± 1 mm. Finally, a 15 ± 1 mm-deep notch was introduced at the bottom center of each SCB specimen to simulate an initial defect and promote crack initiation from the notch tip during loading. The specimen preparation procedure is illustrated in Figure 2. Because laboratory-prepared specimens differ from field-aged materials, aged asphalt mixtures better represent in-service pavement conditions. Accordingly, a two-stage conditioning protocol was adopted, consisting of short-term aging followed by long-term aging. For SBS-modified mixtures, short-term aging was conducted at 135 °C in accordance with R 30 [24]. However, for HVE composite modified mixtures, 135 °C alone may not adequately represent field conditions because of their higher paving and compaction temperatures; therefore, an additional short-term aging temperature of 175 °C was introduced to better simulate field practice. Long-term aging followed AASHTO R 30. Briefly, the loose mixture was heated and mixed uniformly, then spread onto metal trays at an areal density of approximately 21–22 kg/m^2^. The loose mixture was placed in a forced-draft oven for 2 h for thermal-oxidative conditioning, and the material was stirred every hour to ensure uniform aging. After short-term aging, standard specimens were fabricated from the loose mixture using a Superpave gyratory compactor. The specimens were conditioned in a forced-draft oven at 85 °C for 3, 5, and 7 days and then cooled to room temperature for 24 h. The aged specimens were subsequently sectioned using a precision saw, and SCB testing was conducted in accordance with R121 [25]. These conditioning durations have been reported to approximately correspond to 3–10 years of field aging. The same procedure was applied to both SBS-modified mixtures and HVE composite modified mixtures.

Before testing, specimens were conditioned at the target temperature in an environmental chamber for 4 h. SCB tests were performed using a UTM-30 servo-hydraulic testing system. The span-to-diameter ratio was set to 0.8. Prior to loading, a seating load of 0.1 kN (±10 N) was applied at 0.05 kN/s, after which monotonic loading was applied at a crosshead rate of 50 ± 10 mm/min. The test was terminated when the load dropped below 0.1 kN, and load–displacement responses were recorded for each specimen.

**Figure 3:**
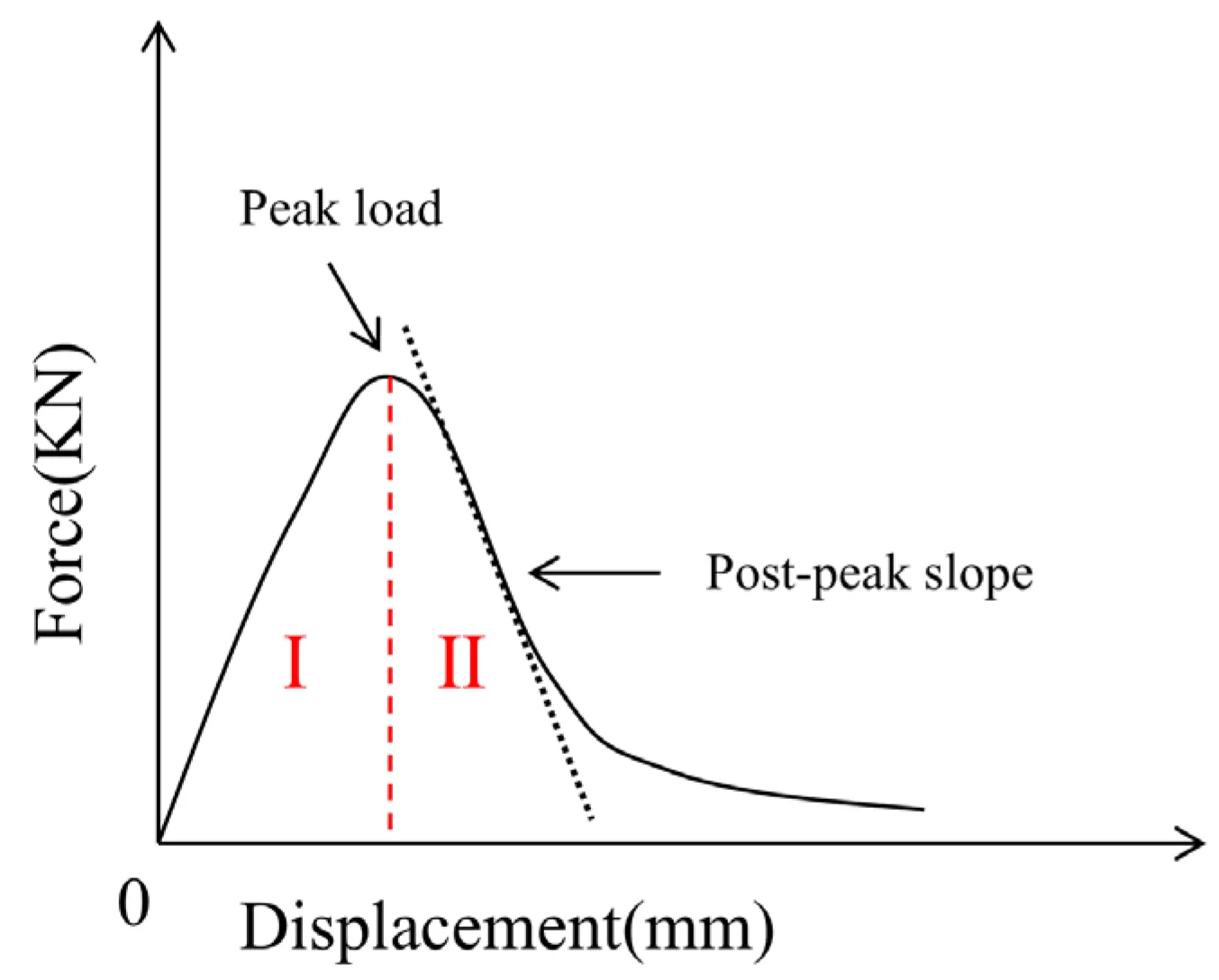
Force-displacement curve diagram. (I) Deformation-dominated stage (II) Crack-propagation-dominated stage.

Figure 3 presents a representative SCB specimen and the corresponding cracking process. The cracking process can be divided into two stages: deformation-dominated response (Stage I) and crack-propagation-dominated response (Stage II). Stage I extends from loading initiation to the peak load, during which microcracks develop and coalesce as the load increases. A visible crack typically forms near the peak load, and the response is dominated by elastic deformation. Stage II is characterized by crack propagation; once the crack reaches a critical length, the load-carrying capacity drops rapidly, leading to failure. Key parameters were extracted from the load–displacement response to calculate cracking indices.

### 3.2. Control Group Protocol

As asphalt is a typical viscoelastic material with high temperature sensitivity, three test temperatures—low (15 °C), medium (25 °C), and high (35 °C)—were set to evaluate temperature effects on cracking in ultra-thin overlay asphalt mixtures. SMA mixtures with three nominal maximum aggregate sizes (NMAS) of 4.75, 8.0, and 9.5 mm were investigated. Two binders were used: a HVE composite modified asphalt and an SBS-modified asphalt, to examine the effect of HVE modification on mixture crack resistance. Three conditioning states were considered: unaged, short-term aged, and long-term aged. Short-term aging was conducted at 135 °C and 175 °C, whereas long-term aging was performed at 85 °C for 0, 3, 5, and 7 days to represent different aging stages over the service life of ultra-thin overlays. Specifically, the 175 °C short-term aging condition was applied exclusively to the HVE mixtures, considering their relatively higher field mixing and compaction temperatures. In contrast, the SBS mixtures were conditioned only at 135 °C. The experimental matrix consisted of 126 combinations, with three replicates tested for each combination, resulting in a total of 378 specimens. The detailed experimental design is summarized in Table 6.

### 3.3. Cracking Performance Indices

#### 3.3.1. Fracture Energy

Fracture energy (*G_f_*) was introduced by the University of Illinois team as part of the development of the Semi-Circular Bending–Illinois Flexibility Index Test (SCB-IFIT) [26]. It quantifies the energy dissipated from loading through crack growth to failure as the area under the load–displacement curve, and it serves as a fundamental index for characterizing mixture cracking resistance in SCB testing, and it is calculated using Equation (1):

(1) $$G_{f}=\frac{W_{f}}{A}$$

where *G_f_* is the fracture energy of the SCB specimen (J/m^2^), *W_f_* is the work of fracture (J), and *A* is the ligament area (m^2^).

#### 3.3.2. Flexibility Index

The flexibility index (*FI*) characterizes the cracking response of asphalt mixtures not only in terms of energy absorption and dissipation, but also by reflecting the evolution of ductility and toughness after crack initiation [27]. FI is sensitive to post-peak softening during crack growth, and it is calculated using Equation (2):

(2) $$FI=\frac{G_{f}}{\left|m\right|}\times 0.01$$

where *G_f_* is the fracture energy of the SCB asphalt mixture specimen (J/m^2^), and *|m|* is the absolute value of the slope at the inflection point of the post-peak load–displacement curve (kN/mm).

#### 3.3.3. Cracking Resistance Index

The Cracking Resistance Index (*CRI*) reduces the variability associated with the Flexibility Index (*FI*) by replacing the post-peak slope at the inflection point (|m|) with the peak load (*|P_max_|*) in the index formulation [9]. *CRI* is intended to characterize the cracking resistance of asphalt mixtures during crack initiation and subsequent crack propagation, as given in Equation (3):

(3) $$CRI=\frac{G_{f}}{\left|P_{\max}\right|}$$

where *G_f_* is the fracture energy of the SCB asphalt mixture specimen (J/m^2^), and *|P_max_|* is the peak load obtained from the load–displacement curve (kN).

### 3.4. Regression-Based Predictive Models and Evaluation

#### 3.4.1. Multiple Linear Regression Model

Multivariate linear regression (MLR) was employed to model the linear relationship between a continuous response variable and two or more explanatory variables, as given in Equation (4). Model parameters were estimated using the ordinary least squares (OLS) method, and the coefficient estimator is given in Equation (5). The sign and magnitude of the regression coefficients indicate the direction and strength of the marginal effect of each predictor on the response. To facilitate comparison among predictors with different units, standardized variables were further used to obtain standardized regression coefficients, which were then used to rank the relative contributions of the influencing factors. Model goodness-of-fit was quantified using the coefficient of determination (R^2^); the statistical significance of individual coefficients was assessed using *t*-tests, and the overall model significance was evaluated using an F-test.

(4) y=Xβ+ε

where *y* represents the measured response, *X* is the matrix of explanatory variables, *β* is the vector of regression coefficients, and *ε* is the error term accounting for unexplained variability.

(5) $$\hat{\beta }={\left(X^{T}X\right)}^{-1}X^{T}y$$

where $\hat{\beta }$ is the coefficient estimate obtained by ordinary least squares, $X^{T}$ denotes the transpose of X, and $(X^{T}X{)}^{-1}$ is the inverse of $X^{T}X$.

#### 3.4.2. Random Forest Regression Model

**Figure 4:**
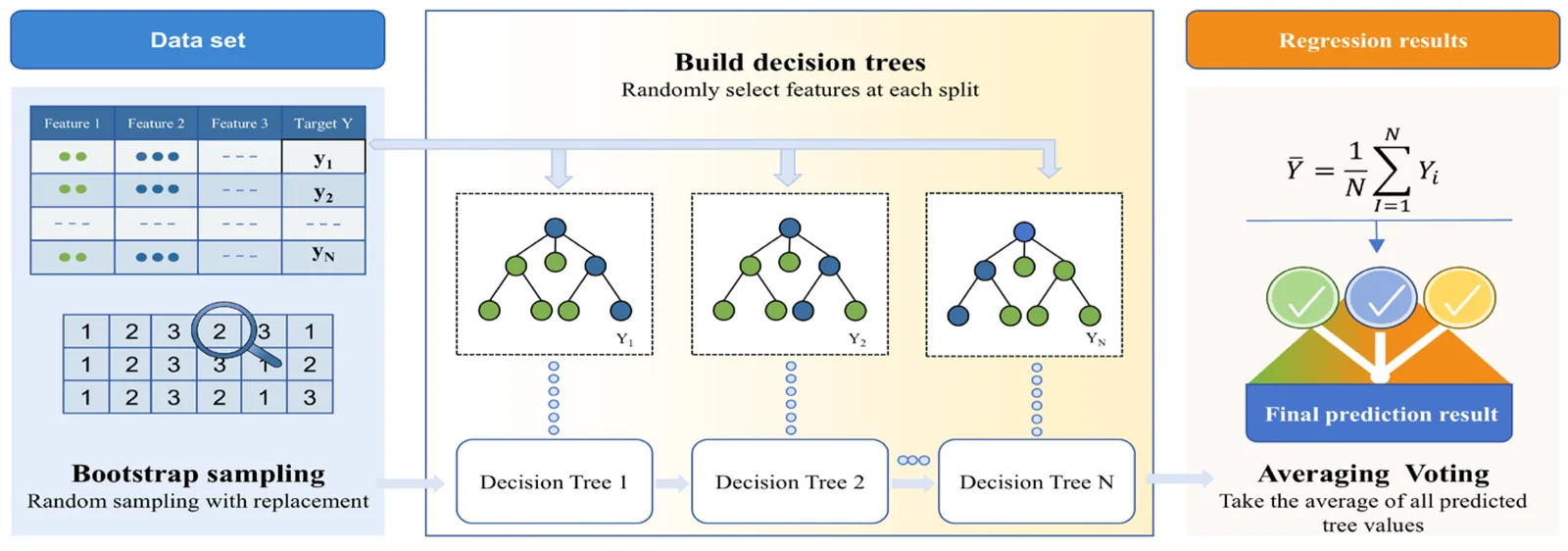
Schematic diagram of random forest regression model.

Random forest regression (RFR) is a tree based ensemble method that combines many regression trees into a single predictor. Figure 4 illustrates the model training process. The training samples for each tree are obtained by bootstrap resampling with replacement from the training set, as shown in Equation (6), and a random subset of features is considered at each split to promote diversity across trees, as shown in Equation (7) [28]. Forest prediction is obtained by averaging the outputs of all trees, as shown in Equation (8). This design reduces the variance of individual trees and improves generalization, and it does not rely on the normality or linearity assumptions of classical regression [29]. RFR is therefore suitable for multi factor problems where nonlinear effects and interactions are expected. Model interpretation can be supported by feature contribution measures such as impurity-based importance, permutation importance, and SHAP values.

(6) $$D^{\left(b\right)}={\left\{(x_{i_{k}},y_{i_{k}})\right\}}_{k=1}^{n},\;i_{k}\in \{1,2,\ldots ,n\}$$

where $D^{\left(b\right)}$ is the bootstrap training set used to fit the b-th tree, $\{(x_{i_{k}},y_{i_{k}}){\}}_{k=1}^{n}$ denotes n samples drawn from the original dataset, and $i_{k}\in \{1,2,\ldots ,n\}$ indicates the index of the selected observation (sampling with replacement).

(7) $$S_{t}\subset \{1,2,\ldots ,p\},\;|S_{t}|=m_{try}$$

where $S_{t}$ is the subset of candidate features randomly selected at node t, p is the total number of available features, and $|S_{t}|=m_{try}$ means that only $m_{try}$ features are considered when searching for the best split at that node.

(8) $$\hat{y}(x)=\frac{1}{N}\sum_{i=1}^{N}y_{i}(x)$$

where $\hat{y}(x)$ is the random-forest prediction for an input x, N is the number of trees in the forest, and $y_{i}(x)$ is the prediction produced by the i-th regression tree; the final output is obtained by averaging the predictions of all trees.

#### 3.4.3. Artificial Neural Network Regression Model

**Figure 5:**
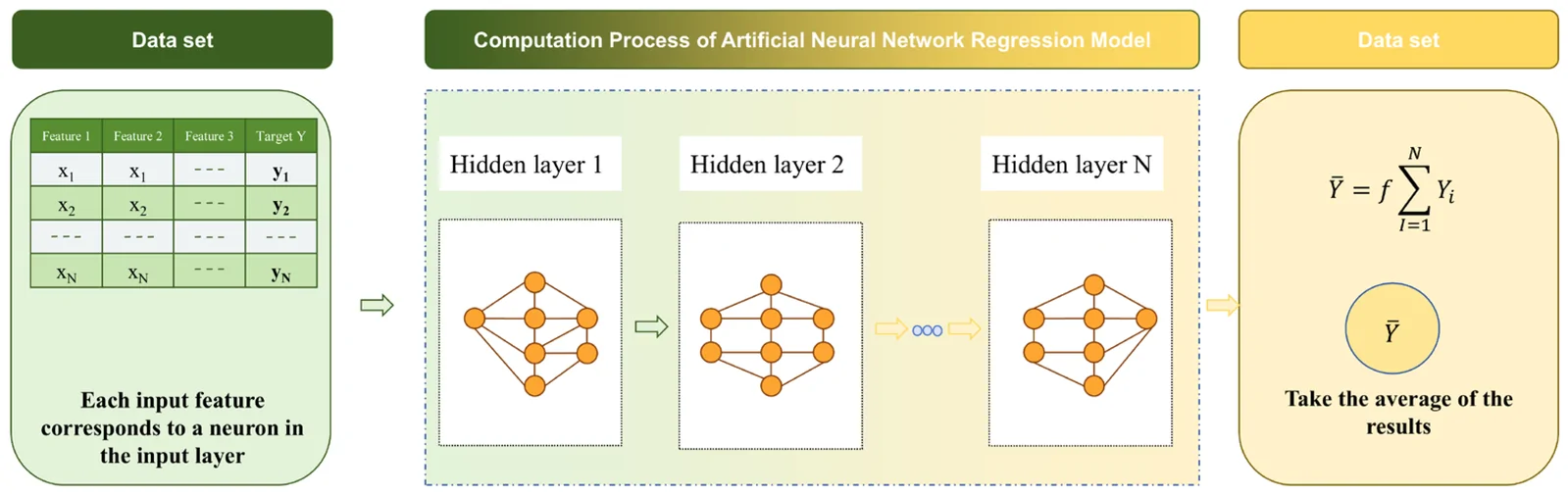
Schematic diagram of artificial neural network regression model.

Artificial neural network regression (ANN) is a bio-inspired nonlinear modeling approach. Its basic unit is the neuron, and interconnected neurons form a network that performs weighted computations and nonlinear transformations [30]. A typical ANN consists of an input layer, one or more hidden layers, and an output layer as shown in Figure 5. The input layer receives the feature vector. The hidden layers apply successive weighted summations and nonlinear activation functions to learn latent representations and approximate the nonlinear mapping from inputs to outputs [31], as expressed in Equation (9). The output layer provides the prediction of the target variable. During training, a loss function is used to measure the discrepancy between predicted and measured values, and the weights and biases are updated iteratively through gradient based backpropagation to minimize the loss [32], as shown in Equation (10). ANN can represent higher-order nonlinear relationships and variable interactions, but its performance depends on the network architecture and hyperparameter settings. For this reason, feature standardization, regularization, and cross validation are adopted to limit overfitting and improve generalization.

(9) $$\begin{array}{l} a^{\left(0\right)}=x\\ z^{\left(l\right)}=W^{\left(l\right)}a^{\left(l-1\right)}+b^{\left(l\right)}\\ a^{\left(l\right)}=\phi \left(z^{\left(l\right)}\right)\\ \hat{y}=a^{\left(L\right)} \end{array}$$

where x is the input feature vector, $W^{\left(l\right)}$ and $b^{\left(l\right)}$ are the weight matrix and bias vector of layer l, ϕ(⋅) is the activation function, $a^{\left(l\right)}$ is the layer output after activation, L is the total number of layers, and $\hat{y}$ is the predicted output.

(10) $$\begin{array}{l} W^{\left(l\right)}\leftarrow W^{\left(l\right)}-\eta \frac{\partial L}{\partial W^{\left(l\right)}},\\ \;b^{\left(l\right)}\leftarrow b^{\left(l\right)}-\eta \frac{\partial L}{\partial b^{\left(l\right)}} \end{array}$$

where η is the learning rate, and $\partial L/\partial W^{\left(l\right)}$ and $\partial L/\partial b^{\left(l\right)}$ are the gradients of the loss with respect to the weights and biases of layer l, computed via backpropagation.

## 4. Results and Discussion

### 4.1. Repeatability and Variability of SCB Test Results

To further examine whether the observed differences among factor levels were statistically supported, one-way analysis of variance (ANOVA) was conducted for each influencing factor and each cracking index. The mean value and 95% confidence interval (CI) were also calculated for each factor level. Since the experimental design was partial factorial and unbalanced, the ANOVA results were used as supporting statistical evidence for the observed trends rather than as definitive proof of independent causal effects. As shown in Table 7, temperature, NMAS, asphalt type, short-term aging condition, and long-term aging duration all showed statistically significant differences in *G_f_*, *FI*, and *CRI* among the tested combinations (*p* < 0.05), indicating that the observed variations in the cracking indices were statistically supported.

### 4.2. Effect of Temperature on Cracking Indices of Ultra-Thin Overlay Asphalt Mixtures

**Figure 6:**
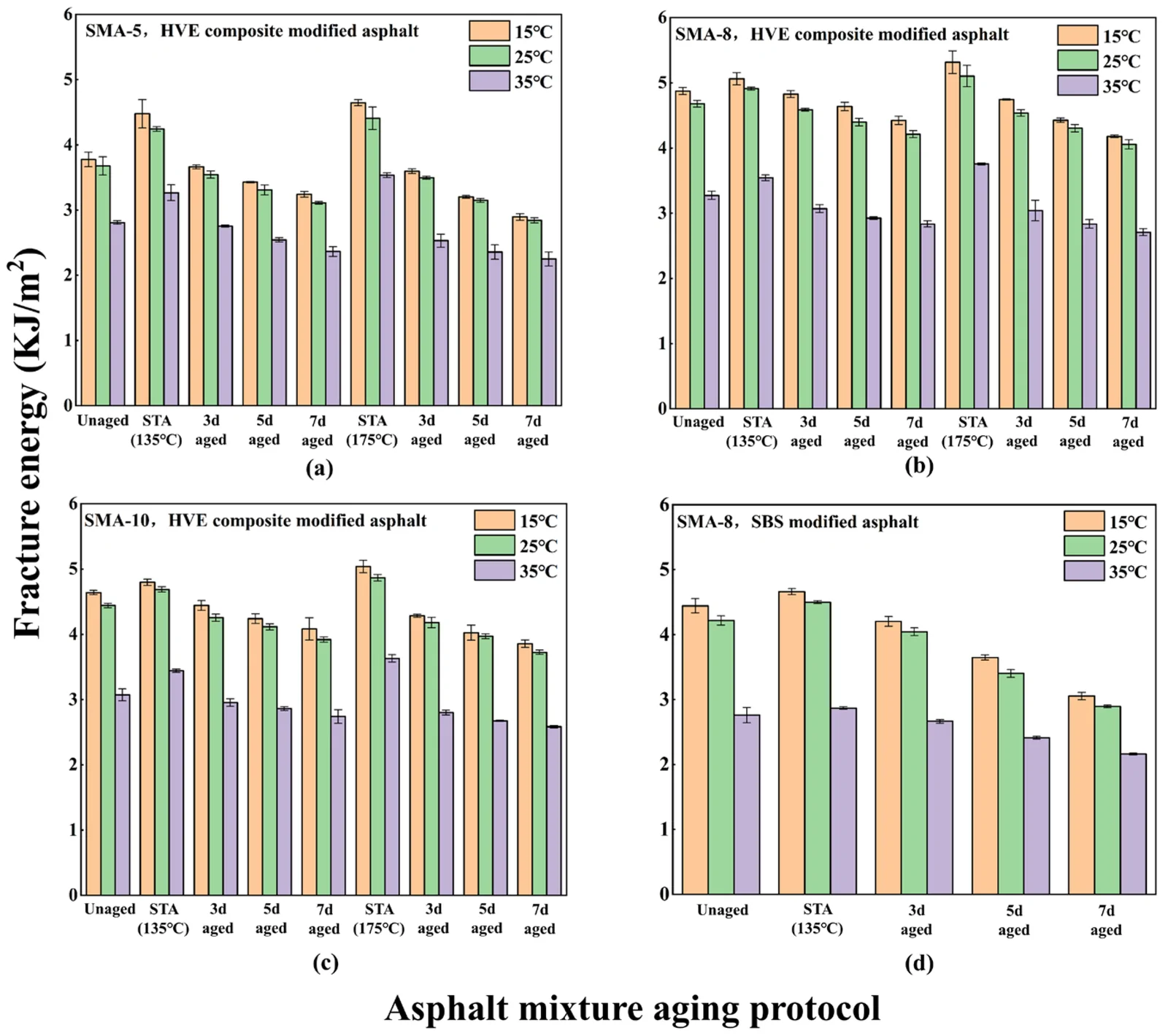
Effect of environmental temperature on *G_f_*. (**a**) SMA-5, HVE composite modified asphalt (**b**) SMA-8, HVE composite modified asphalt (**c**) SMA-10, HVE composite modified asphalt (**d**) SMA-8. SBS modified asphalt.

**Figure 7:**
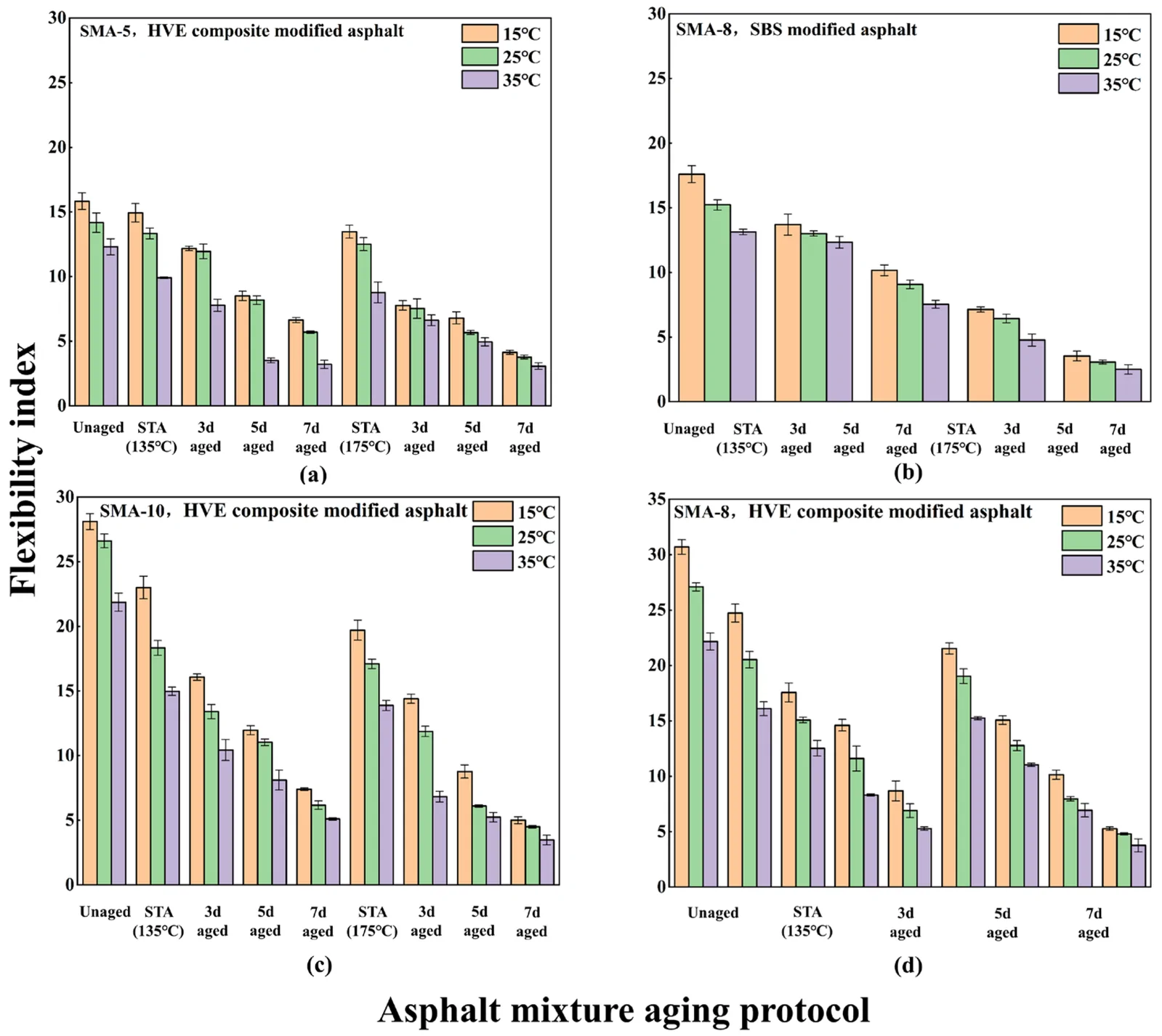
Effect of environmental temperature on *FI*. (**a**) SMA-5,HVE composite modified asphalt (**b**) SMA-8, HVE composite modified asphalt (**c**) SMA-10, HVE composite modified asphalt (**d**) SMA-8. SBS modified asphalt.

**Figure 8:**
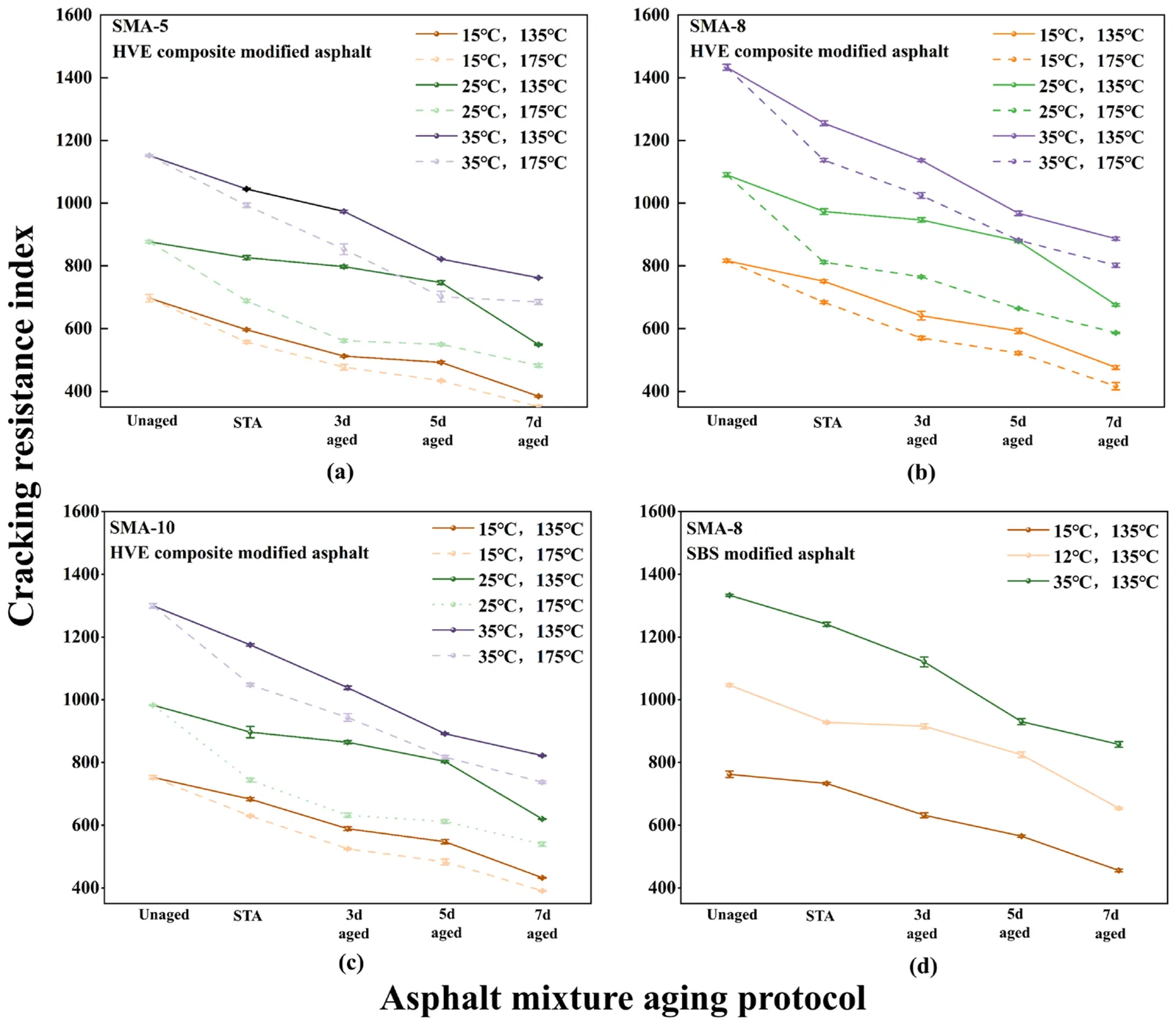
Effect of environmental temperature on *CRI*. (**a**) SMA-5, HVE composite modified asphalt (**b**) SMA-8, HVE composite modified asphalt (**c**) SMA-10, HVE composite modified asphalt (**d**) SMA-8. SBS modified asphalt.

Figure 6, Figure 7 and Figure 8 illustrate the effects of test temperature on *G_f_*, *FI*, and *CRI*, respectively. For the four mixtures (SMA-5/HVE, SMA-8/HVE, SMA-10/HVE, and SMA-8/SBS), *G_f_* and FI reached their highest values at the low temperature and decreased as temperature increased. This result is associated with the higher binder viscosity and mixture stiffness at a low temperature. These changes increase the peak load and the pre-peak load-carrying capacity, which leads to a higher *G_f_*. When temperature increased from the low to the high level, the average change rates of *G_f_* were −25.86%, −34.27%, −32.25%, and −35.23% for SMA-5/HVE, SMA-8/HVE, SMA-10/HVE, and SMA-8/SBS, respectively. Notably, the reduction from the intermediate to the high temperature accounts for approximately 90% of the overall decrease, indicating that the decline in *G_f_* is concentrated in the higher-temperature range. As temperature increases further, the magnitude of *G_f_* reduction becomes markedly larger, suggesting a more pronounced loss of fracture energy dissipation capacity under high-temperature conditions. *FI* is a composite index that reflects both fracture energy and post-peak softening. A lower temperature may produce a steeper post-peak drop. However, the increase in *G_f_* dominates the numerical response, and *FI* therefore remains higher at low temperature. This behavior indicates higher overall energy dissipation and greater resistance to crack growth during the propagation stage. The average change rates of *FI* were −33.28%, −32.21%, −34.28%, and −24.74%. The last group shows a smaller change rate than the other three, whereas the first three groups exhibit similar change rates. This comparison suggests that asphalt type affects the temperature sensitivity of *FI*. The SBS-modified mixture shows a lower temperature sensitivity, but its *FI* remains lower than that of the HVE-modified mixtures under comparable conditions.

In contrast to *G_f_* and *FI*, *CRI* increased with temperature. The average growth rates were 78.91%, 75.21%, 75.36%, and 72.78%. Among the three indices, *CRI* showed the strongest temperature sensitivity. Meanwhile, its trend was relatively consistent across mixtures. The opposite trend is likely related to the formulation of CRI. Within 15–35 °C, both the peak load and G_f_ decreased as temperature increased. However, according to the CRI calculation, the attenuation rate of G_f_ was lower than that of the peak load. In other words, the denominator decreased more rapidly than the numerator, leading to an increase in the calculated CRI. Therefore, this increase does not indicate an improvement in absolute strength or load-bearing capacity, but reflects the relatively slower degradation of fracture energy compared with peak load. This suggests that, within an intermediate temperature range, CRI may be less effective in capturing strength degradation and may lead to biased interpretation in some cases.

### 4.3. Effect of Asphalt Type on Cracking Indices of Ultra-Thin Overlay Asphalt Mixtures

**Figure 9:**
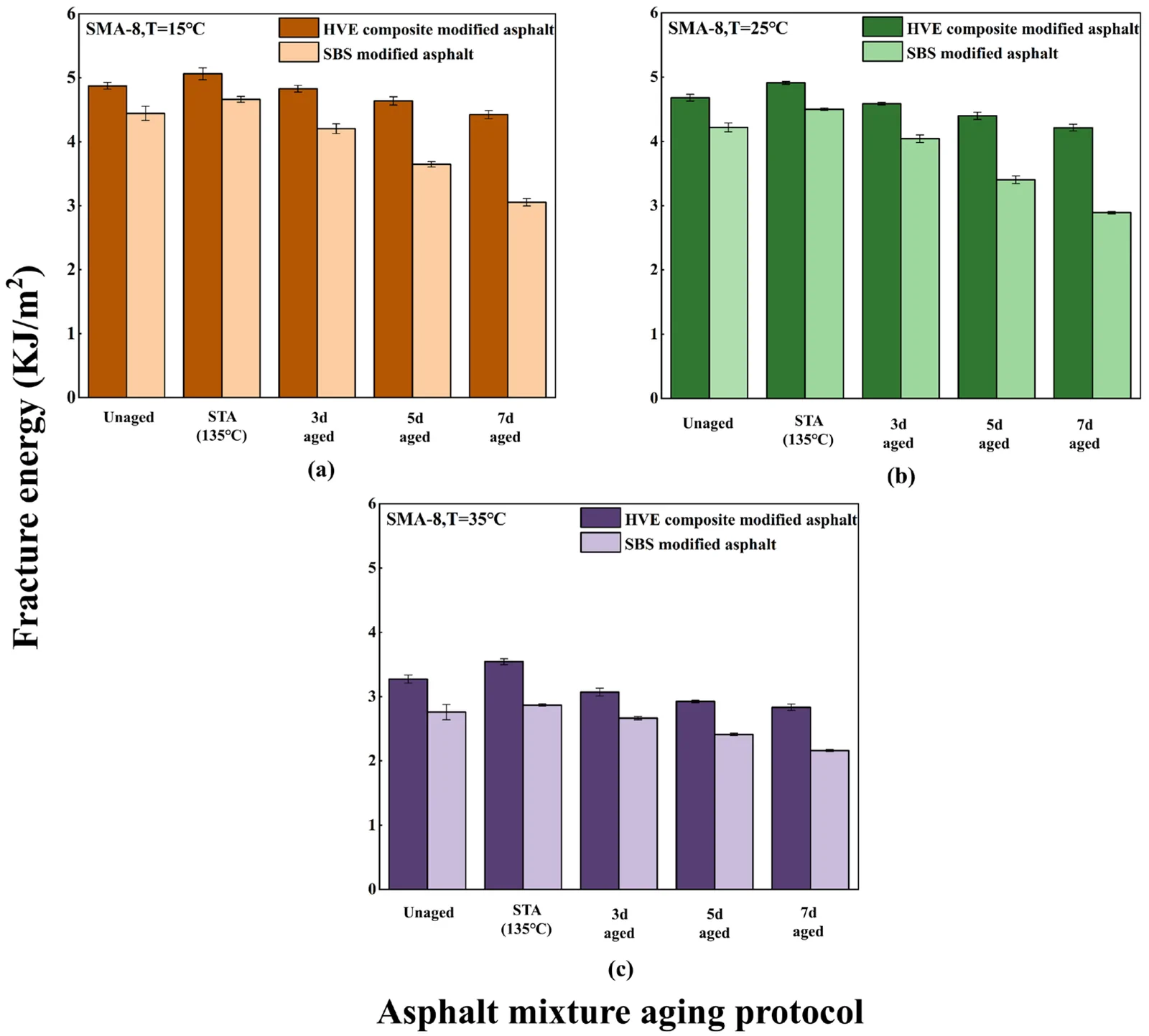
Effect of asphalt type on *G_f_*. (**a**) SMA-8, 15 °C (**b**) SMA-8, 25 °C (**c**) SMA-8, 35 °C.

**Figure 10:**
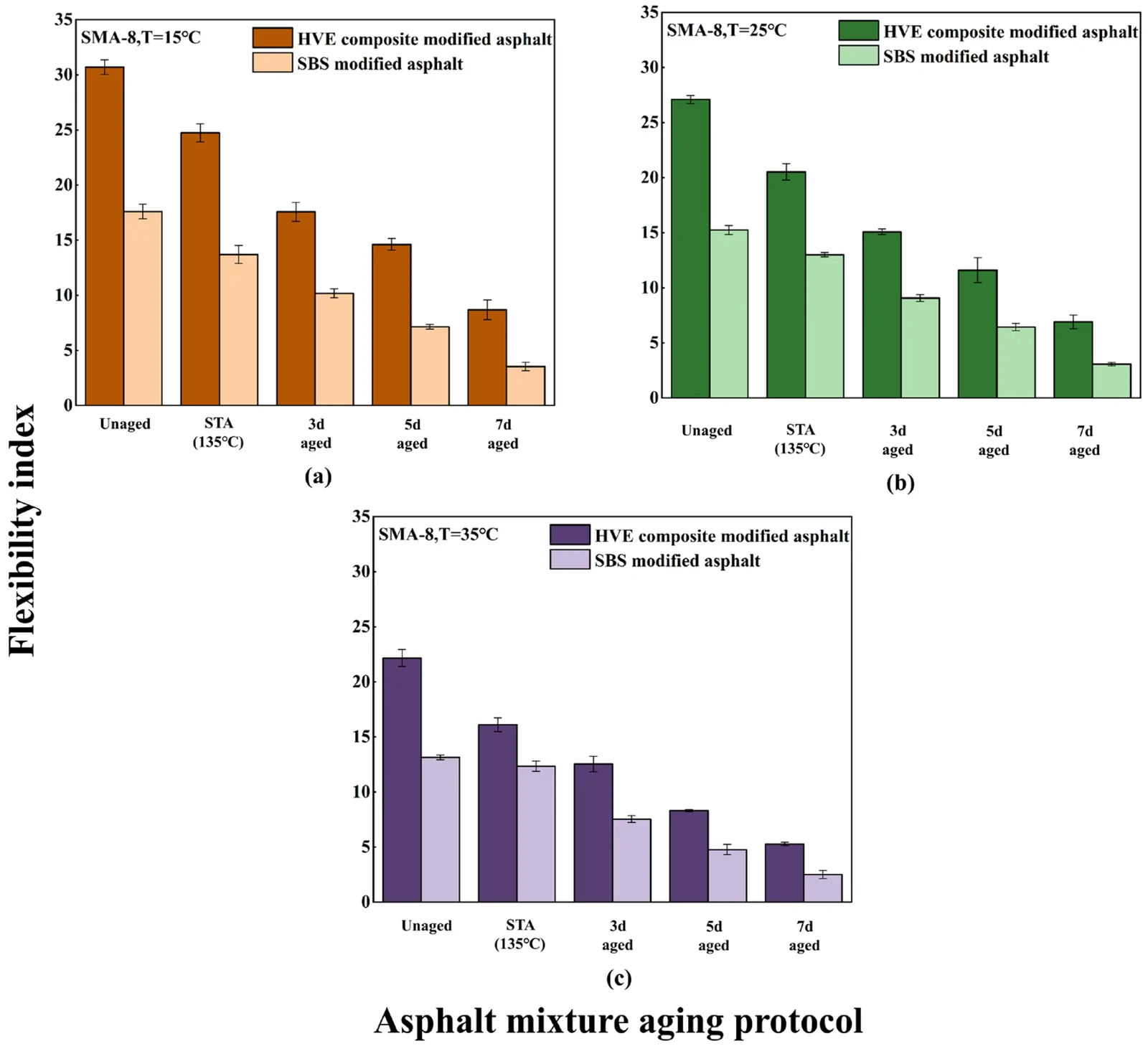
Effect of asphalt type on *FI*. (**a**) SMA-8, 15°C (**b**) SMA-8, 25°C (**c**) SMA-8, 35 °C.

**Figure 11:**
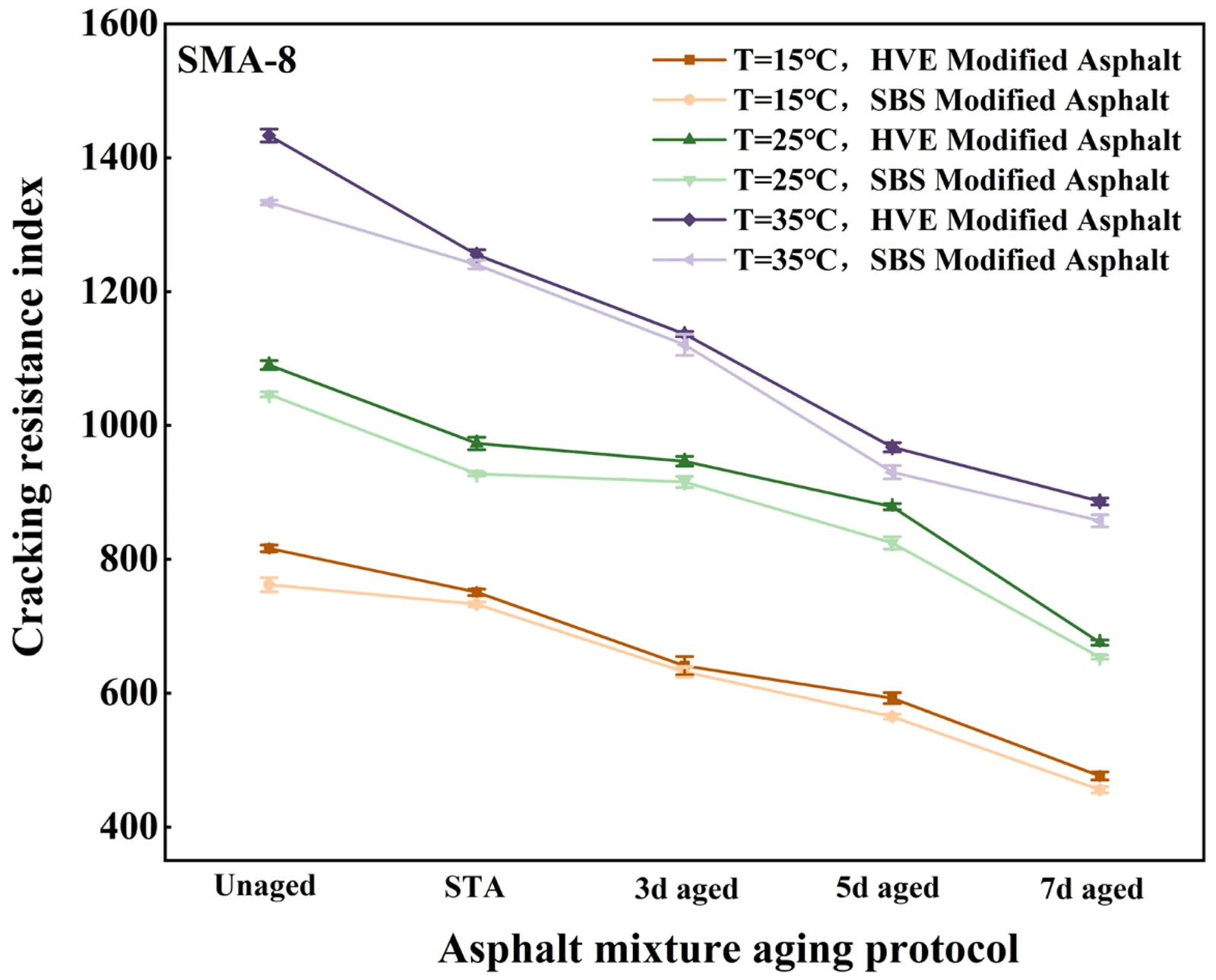
The effect of asphalt type on cracking resistance index.

Figure 9, Figure 10 and Figure 11 present the effects of the HVE modifier on the cracking-related indices. The overall trends are similar across mixtures with different NMAS. Therefore, to avoid repetitive discussion, the SMA-8 mixture is used as a representative case for detailed analysis. For the SMA-8 mixture, the average *G_f_* of the HVE composite-modified asphalt increases by 16.41%, 16.81%, and 17.90% compared with that of the SBS-modified asphalt. This indicates that within the scope of this study, HVE-modified asphalt exhibits superior aging resistance compared to SBS-modified asphalt. At 15 °C and 25 °C, the performance gap increases with aging duration and reaches a maximum of 30.99% and 31.33%, respectively. The results indicate that the HVE modifier provides only a limited improvement in fracture energy at low temperatures, and the two materials exhibit comparable fracture energy at the early stage of aging. As aging progresses, however, the HVE system shows a stronger ability to retain fracture energy, demonstrating superior long-term stability. In contrast, no similar stage-dependent divergence is observed at higher temperature; the performance gap varies only slightly with aging time, and HVE mainly exhibits a stable and sustained advantage. At 15 °C, 25 °C, and 35 °C, the mean reduction in *FI* was 47.96%, 44.08%, and 39.83%, respectively. The corresponding mean reduction in *CRI* was 2.77%, 4.39%, and 3.34%, respectively. These results indicate that the improvement in cracking performance is mainly associated with post-peak toughening. The modifier enhances the energy dissipation capacity and resistance to crack growth. In contrast, its contribution to the ultimate load-carrying capacity at crack initiation appears limited. From a binder perspective, the HVE modifier improves viscoelastic toughness, ductility, and stress-relaxation capability. This allows the crack-tip region to accommodate larger deformation and dissipate more energy, thereby delaying crack propagation.

### 4.4. Effect of NMAS on Cracking Indices of Ultra-Thin Overlay Asphalt Mixtures

**Figure 12:**
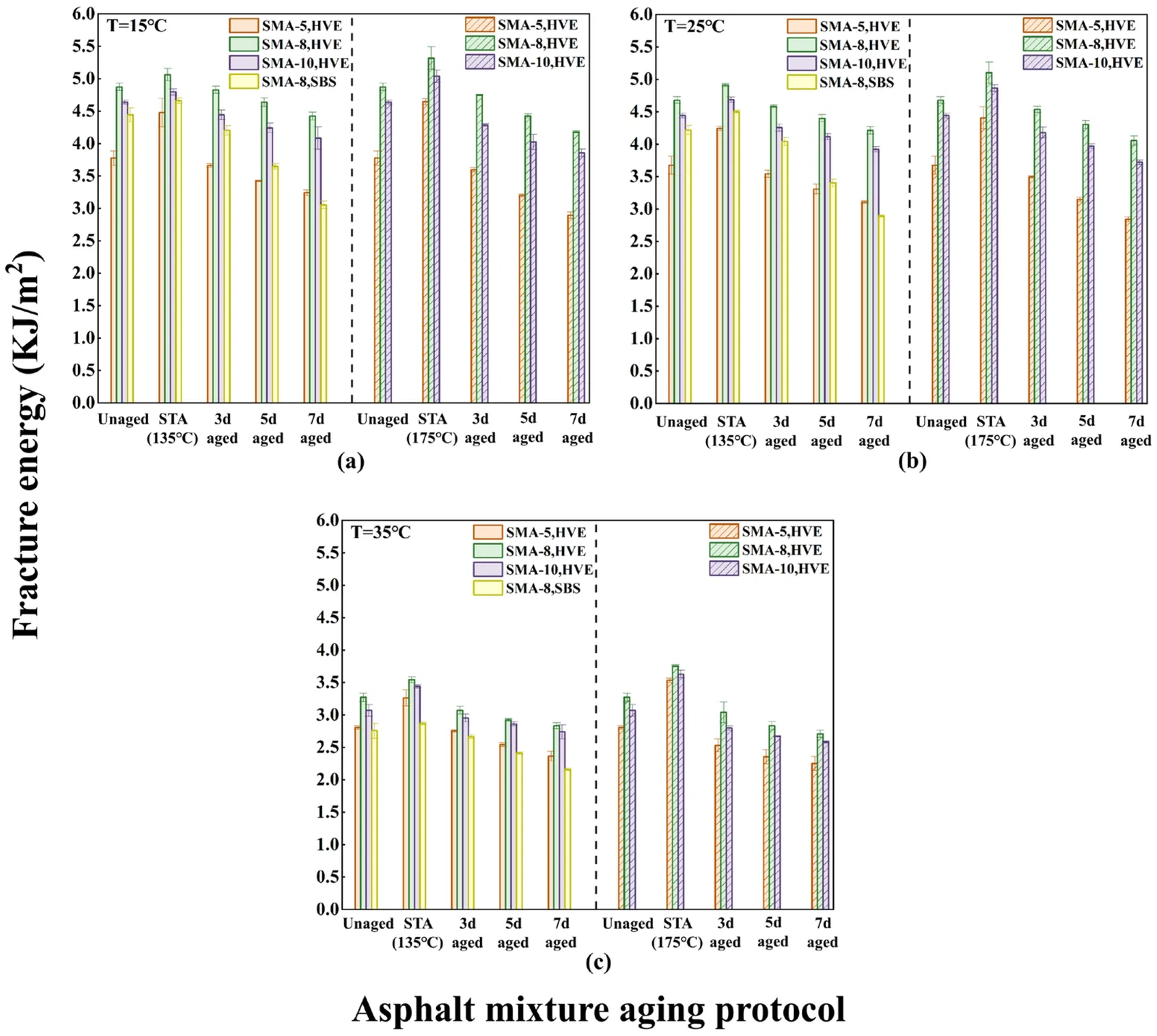
Effect of nominal maximum aggregate size on *G_f_*. (**a**) 15 °C (**b**) 25 °C (**c**) 35 °C.

**Figure 13:**
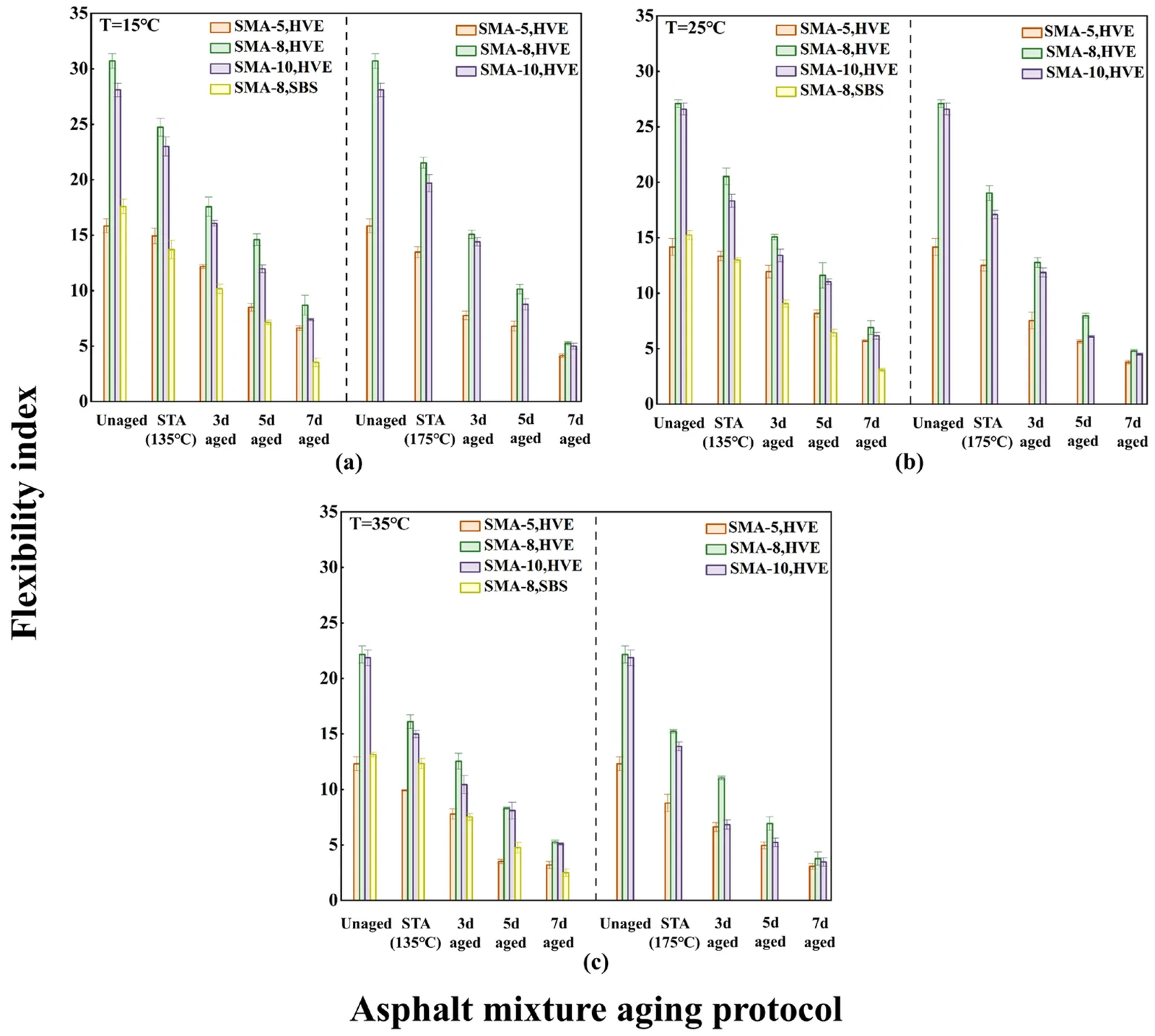
Effect of nominal maximum aggregate size on *FI*. (**a**) 15 °C (**b**) 25 °C (**c**) 35 °C.

**Figure 14:**
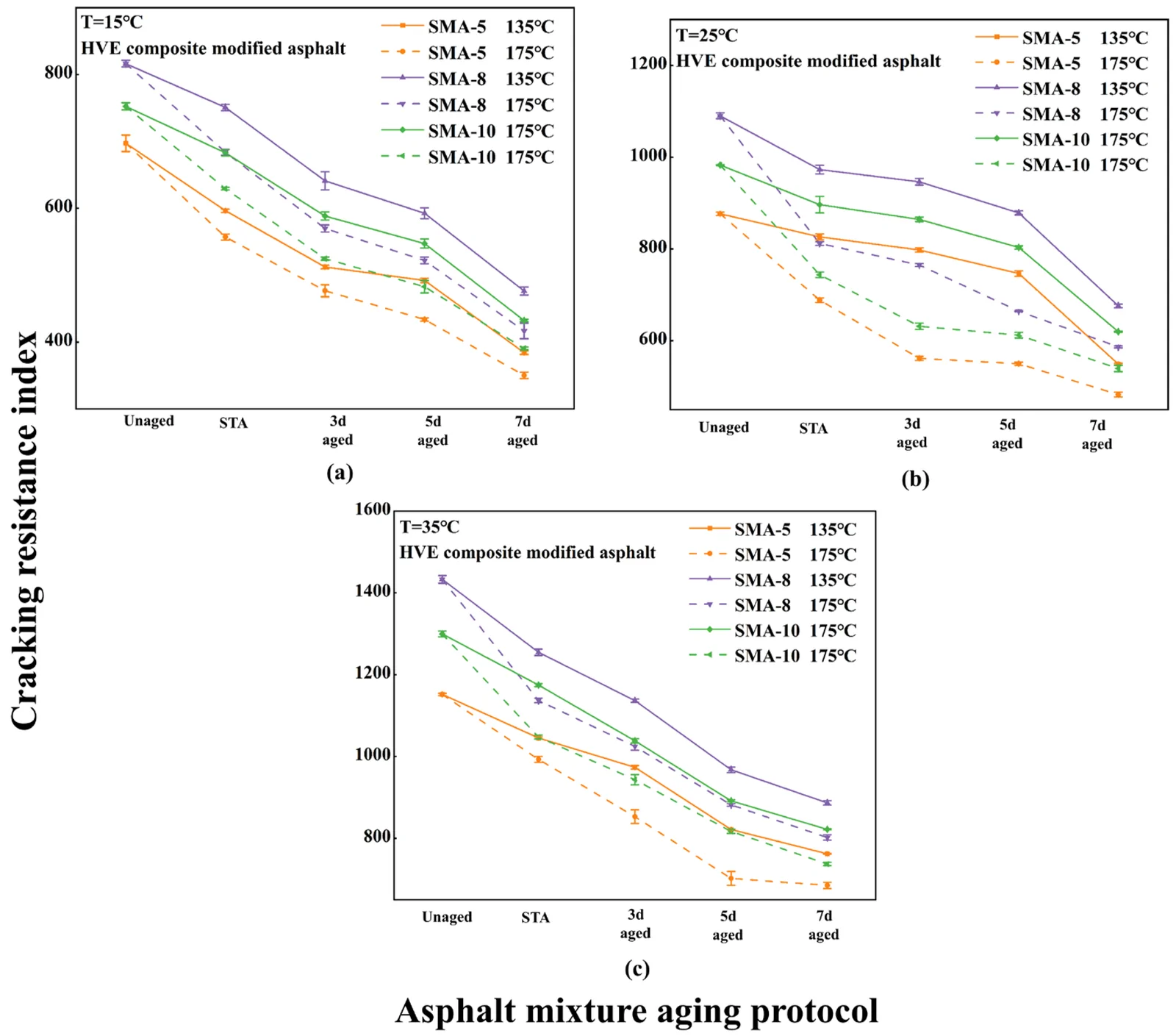
Effect of nominal maximum aggregate size on CRI. (**a**) 15 °C, HVE composite modified asphalt (**b**) 25 °C, HVE composite modified asphalt (**c**) 35 °C, HVE composite modified asphalt.

Figure 12, Figure 13 and Figure 14 show that *G_f_*, *FI* and *CRI* increase first and then decrease as the nominal maximum aggregate size (NMAS) increases. This trend suggests that the coarse aggregate skeleton in the fine-graded mixture (SMA-5) provides limited load-bearing and load-transfer capacity. Consequently, the mixture becomes more vulnerable to external loading. In addition, a more uniform aggregate structure reduces crack-path tortuosity during propagation. This provides less resistance to crack growth and can accelerate crack development. Overall, SMA-5 shows the lowest values across the three indices, indicating the weakest overall cracking resistance. Compared with SMA-8, the coarser mixture (SMA-10) does not provide superior cracking resistance. Instead, *G_f_*, *FI*, and *CRI* all decrease. This may be attributed to the increased demand for intermediate-size aggregates (3–5 mm) to maintain skeleton stability in SMA-10. A higher proportion of the 3–5 mm fraction can disturb the continuity of the interlocking coarse skeleton. It also reduces the effective asphalt film thickness and weakens the ductile energy dissipation provided by the rich mastic. As a result, cracks can penetrate more readily, crack-growth resistance decreases, and failure occurs earlier.

Notably, the NMAS-induced variation in *G_f_* at 15 °C and 25 °C is much larger than that at 35 °C, with a magnitude of change approximately twice that observed at 35 °C. The average change rates for the SMA-5–SMA-8 increment, SMA-8–SMA-10 increment, and the overall SMA-5–SMA-10 increment are 30.39%, −7.09%, and 21.05% at 15 °C; 29.30%, −7.38%, and 20.96% at 25 °C; and 15.56%, −4.63%, and 10.16% at 35 °C. This phenomenon is likely associated with the higher binder stiffness at low temperature. At low temperature, the binder becomes stiffer and interacts more effectively with aggregates to carry greater loads, which amplifies the NMAS-related differences in *G_f_*. As temperature increases, binder viscosity decreases and the mixture becomes more compliant. Consequently, the load-carrying contribution of the binder is reduced, and the NMAS-related contrast becomes less pronounced.

### 4.5. Effect of Aging on Cracking Indices of Ultra-Thin Overlay Asphalt Mixtures

**Figure 15:**
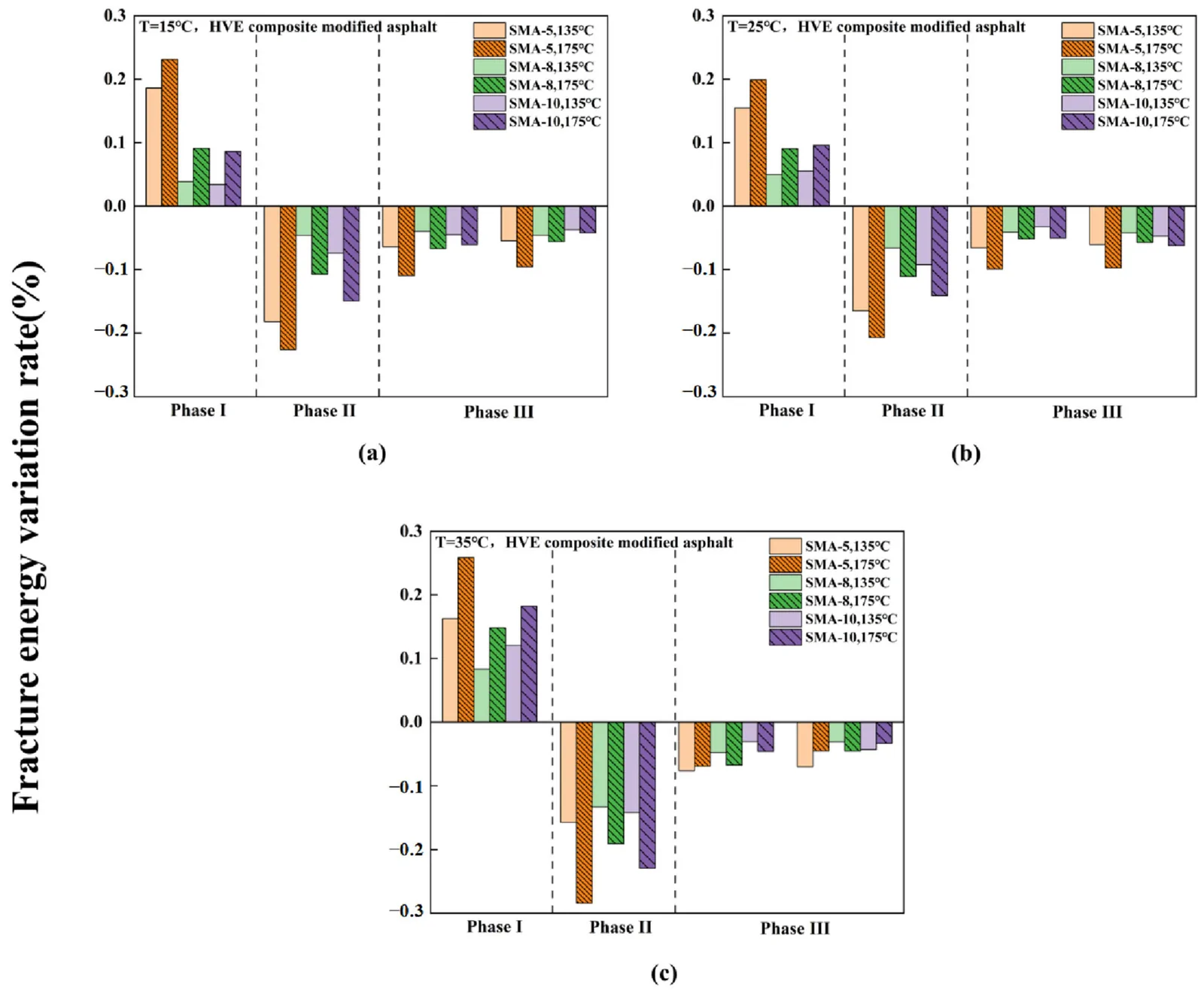
Effect of aging on the rate of change in *G_f_*. (**a**) T = 15 °C, HVE composite modified asphalt; (**b**) T = 25 °C, HVE composite modified asphalt; (**c**) T = 35 °C, HVE composite modified asphalt.

Figure 15 shows the variation in *G_f_* under different aging schemes. Together with the *FI* and *CRI* results in Figure 13 and Figure 14, the aging effect on cracking-related performance of ultra-thin overlay mixtures exhibit a clear staged evolution that can be divided into three phases. Phase I corresponds to short-term aging. After short-term aging, *G_f_* increases relative to the unaged condition, whereas *FI* and *CRI* show an overall downward shift, suggesting that post-peak-related responses are more sensitive to aging. Accordingly, short-term aging leads to opposite trends between the total energy-related index *G_f_* and the post-peak-related indices *FI* and *CRI*. Compared with short-term aging at 135 °C, short-term aging at 175 °C results in a more pronounced increase in *G_f_*, with an increase of approximately 0.28 to 1.54 times. Phase II represents the early stage of long-term aging, around 3 d. The short-term-aging benefit in *G_f_* largely disappears, and *G_f_* drops rapidly to below the unaged level. Meanwhile, *FI* and *CRI* continue to decrease markedly, indicating a pronounced deterioration in cracking-related performance. Phase III corresponds to advanced long-term aging, from 5 to 7 d. *FI* and *CRI* keep decreasing, while *G_f_* continues to decline with a gradually reduced rate of change, indicating a downward tendency that becomes less steep at the late aging stage. For *FI* and *CRI*, specimens subjected to 175 °C short-term aging generally deteriorate faster than those aged at 135 °C at each stage, indicating that a higher short-term aging temperature accelerates damage and weakens the cracking resistance of the asphalt mixture. Overall, the results show that *G_f_* increases after short-term aging, *FI* and *CRI* display more persistent and stable reductions, whereas all three indices exhibit predominantly decreasing trends with prolonged long-term aging.

## 5. Evaluation of Factors Affecting Crack Resistance in Ultra-Thin Surface Course Asphalt Mixtures Based on Multiple Models

Previous studies indicate that cracking indices differ in their sensitivity to influencing factors, so the importance ranking of the same factor may vary across metrics. To enable cross-metric comparison, multiple linear regression, random forest regression, and artificial neural network models were developed using SPSS 27 and PyCharm 2024.2. The dataset included 126 condition combinations with three replicates each, yielding 378 observations. Quantitative variables, including temperature, NMAS, and long-term aging duration, were directly used as numerical inputs, while categorical variables were encoded using a dictionary-based method. In contrast, asphalt type and aged state were treated as discrete categorical factors and encoded using one-hot encoding to avoid imposing artificial ordinal or linear relationships among categorical levels. Specifically, high-viscosity asphalt was used as the reference category for asphalt type, and SBS asphalt was represented by an independent dummy variable. For the aged state, the unaged condition was used as the reference category, while the 135 °C and 175 °C short-term aged states were represented by two separate dummy variables.

Because replicate specimens share identical input features, including temperature, gradation, asphalt type, short-term aging pretreatment status, and long-term aging duration, placing replicates from the same condition in both the training and test sets would introduce information leakage and inflate the estimated generalization performance. To avoid this bias, a group identifier was defined for each condition combination, and GroupShuffleSplit was used to ensure that all replicates from the same condition were always assigned to the same subset.

### 5.1. Recommendations for RFR and ANN Models and Optimization

Hyperparameter optimization is important for machine learning model performance because it controls the behavior of the training algorithm. Hyperparameters for the RFR and ANN models were tuned separately for the three cracking related metrics. GridSearchCV was applied to the training set with five-fold group cross validation using GroupKFold to select the optimal settings and reduce inter group information leakage. The selected hyperparameters are summarized in Table 8 and Table 9. To improve the reproducibility of the ANN modeling procedure, the main training settings were further specified. The number of hidden layers, neurons, activation function, learning rate, and L2 coefficient were treated as tunable hyperparameters, while the other training settings were fixed after preliminary testing. Specifically, the number of epochs was set to 200, and mean squared error was used as the loss function. The batch size was selected between 16 and 32 through grid search. The input variables were standardized using StandardScaler, whereas the output variables were kept in their original scales. A fixed random seed was used, and the optimal ANN model for each target variable was refitted once after five-fold GroupKFold cross-validation and then evaluated on the independent test set.

**Figure 16:**
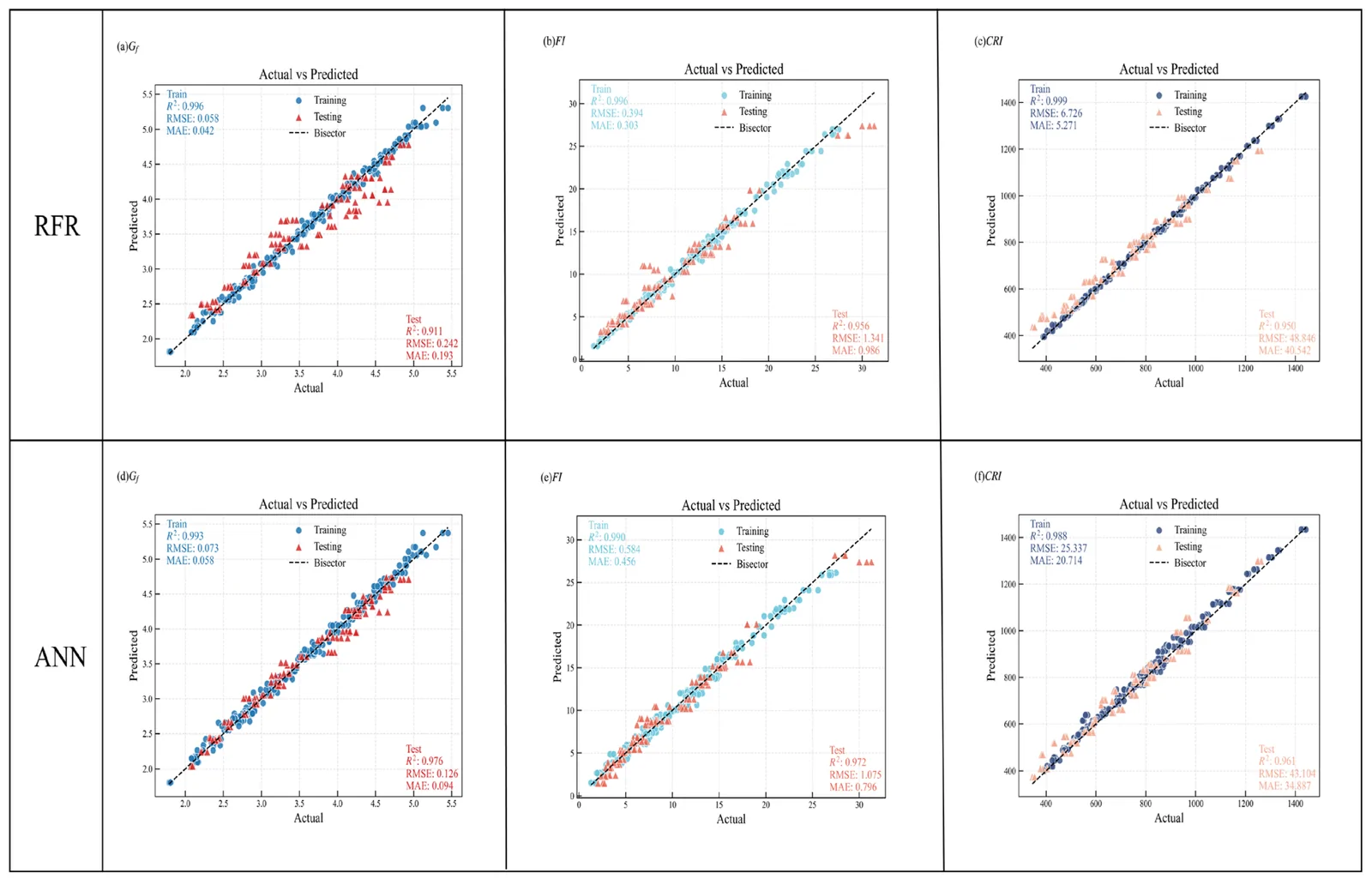
Actual–predicted comparison for RFR and ANN models.

Figure 16 compares predicted versus observed values for the training and test sets, where the 45° line denotes perfect agreement. After hyperparameter tuning, the points concentrate tightly around this line, indicating strong predictive accuracy. Both RFR and ANN maintain high R^2^ across all three metrics, with only a small train–test gap, suggesting no evident over fitting.

### 5.2. Comparison of Accuracy Among Three Models

To compare the fitting performance of the three models, three metrics were used: the coefficient of determination (R^2^), the root mean squared error (RMSE), and the mean absolute error (MAE). R^2^ measures how much of the variance in the response variable is explained by the model relative to a mean-based baseline. A higher R^2^ indicates better explanatory capability. RMSE describes the typical magnitude of the error between predicted and measured values. Because it squares the errors, RMSE penalizes large deviations more strongly and is sensitive to occasional large errors. MAE is the average absolute prediction error and has the same unit as the response variable. It is less affected by outliers than RMSE and reflects the overall prediction bias across the dataset. The definitions of R^2^, RMSE, and MAE are given in Equations (11)–(13). The results are summarized in Table 10. The results are summarized in Table 9, where the hyperparameters were selected by five-fold GroupKFold cross-validation on the training set only, the final model performance was evaluated on a held-out grouped test set.

(11) $$R^{2}=1-\frac{\sum_{i=1}^{n}{\left(y_{i}-{\hat{y}}_{i}\right)}^{2}}{\sum_{i=1}^{n}{\left(y_{i}-\bar{y}\right)}^{2}}$$

(12) $$\mathrm{RMSE}=\sqrt{\frac{1}{n}\sum_{i=1}^{n}{\left(y_{i}-{\hat{y}}_{i}\right)}^{2}}$$

(13) $$\mathrm{MAE}=\frac{1}{n}\sum_{i=1}^{n}\left|y_{i}-{\hat{y}}_{i}\right|$$

where $y_{i}$ denotes the measured value of the i-th sample, ${\hat{y}}_{i}$ denotes the corresponding predicted value, $\bar{y}$ denotes the mean of the measured values, and n denotes the number of samples.

It can be seen that among the three models, MLR exhibits the lowest prediction accuracy. RFR and ANN demonstrate superior fitting performance with smaller errors, indicating their greater ability to characterize nonlinear responses, with ANN showing the best overall performance. Across all target metrics, ANN consistently achieves higher R^2^ values and lower RMSE and MAE. The poor results of MLR suggest that the relationship between inputs and outputs is not purely linear. Nonlinear effects and variable interactions may exist within the data.

### 5.3. Model Interpretability Assessment

To describe the factor effects on cracking indices, the MLR model was interpreted using the sign of the regression coefficients, where positive and negative coefficients indicate positive and negative associations, respectively. For the RFR and ANN models, SHAP summary plots were used for feature-effect interpretation. In the SHAP plots, positive SHAP values indicate an increase in the predicted index, whereas negative values indicate a decrease. The absolute SHAP value reflects the magnitude of the feature contribution, and the color distribution was used to identify increasing, decreasing, or non-monotonic trends. The mean absolute SHAP value, Mean (|SHAP|), was used to rank the overall importance of input variables. SHAP analysis was performed using the model-agnostic KernelExplainer, with up to 60 training samples as the background dataset and up to 120 grouped test samples for explanation. The number of SHAP sampling evaluations was set to 300, and both beeswarm plots and mean (|SHAP|) bar plots were generated.

The MLR results are summarized in Table 11. All factors show statistically significant effects on the cracking resistance indices of the asphalt mixture. Long term aging time has a strong negative effect on *G_f_* and *FI* and is the most influential factor for *FI*. Ambient temperature reduces *G_f_* and FI but increases *CRI*, which reflects that the indices focus on different parts of the cracking response. Asphalt type has a clear effect on *G_f_* and *FI*, while its effect on *CRI* is weaker, indicating that its contribution is more evident in post peak toughness and energy dissipation than in the *CRI* definition.

**Figure 17:**
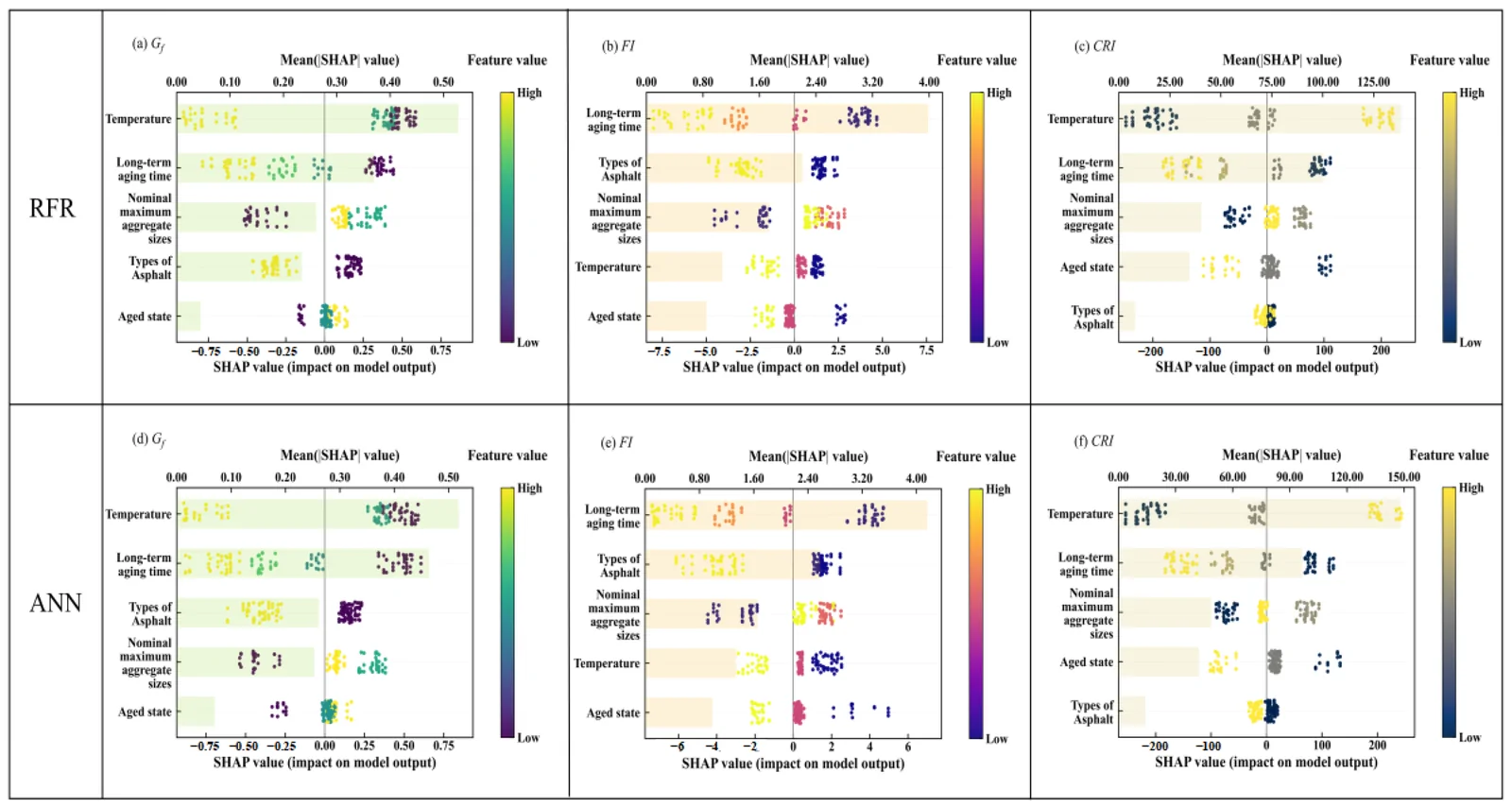
Comparison of SHAP feature importance between RFR and ANN.

Figure 17 presents SHAP summary plots for the RFR and ANN models. Ambient temperature and long term aging time show predominantly negative SHAP values in both models, and higher feature values are associated with larger absolute SHAP values, indicating a consistent decrease in *G_f_* with increasing temperature and aging severity. NMAS and asphalt type span positive and negative SHAP ranges, and higher values more often align with positive SHAP values, indicating a modest increase in *G_f_* and a smaller contribution than temperature and long-term aging. Both models show a non-monotonic NMAS relationship, with Gf increasing initially and then decreasing.

For *FI*, long term aging time shows a consistently negative contribution in both models, indicating progressive loss of post peak toughness during crack propagation with increasing aging. A clear binder-system difference is also observed for FI: mixtures with the HVE composite modified asphalt exhibit overall higher predicted FI values, suggesting a stronger resistance to aging-induced deterioration and better crack-resistance retention than those with the SBS-modified asphalt. Notably, this binder-type contrast is more pronounced in the ANN model, implying that FI is particularly sensitive to the binder’s viscoelastic descriptors and associated nonlinear responses, and may therefore capture the influence of binder modification on crack-growth behavior more effectively than other cracking-related indices.

For *CRI*, ambient temperature shows the strongest positive contribution. High temperature samples have SHAP values concentrated on the positive side with a wider spread. Long term aging time remains a consistently negative contributor, indicating reduced resistance to crack development with increasing aging. Asphalt type and NMAS yield SHAP values closer to zero for *CRI*, indicating smaller contributions. *CRI* shows lower sensitivity to mixture composition and structural parameters and greater sensitivity to environmental conditions.

Overall, the conclusions drawn from MLR differ to some extent from the qualitative patterns discussed above. This discrepancy reflects, at least in part, the inability of linear models to fully capture nonlinear effects and feature interactions, thereby limiting mechanistic interpretability. In contrast, ANN and RFR yield factor-importance rankings and contribution directions that are highly consistent with the observed trends across the three cracking indices. This indicates that, under the data characteristics and experimental design of this study, machine-learning-based analyses provide more robust and consistent qualitative mechanistic insights for identifying dominant factors and supporting mechanism-oriented discussions.

### 5.4. Analysis of Crack Formation Factors

The three modeling approaches differ fundamentally in how they quantify factor contributions. For MLR, standardized regression coefficients (Beta), obtained after feature standardization, represent the linear marginal effects of the predictors on the response and their relative strengths. For RFR and ANN, factor importance is derived from SHAP attributions, using mean (|SHAP|) as an overall contribution measure for ranking. Because these contribution metrics are on different scales across models, all coefficients were further normalized to enable a consistent cross-model comparison.

**Figure 18:**
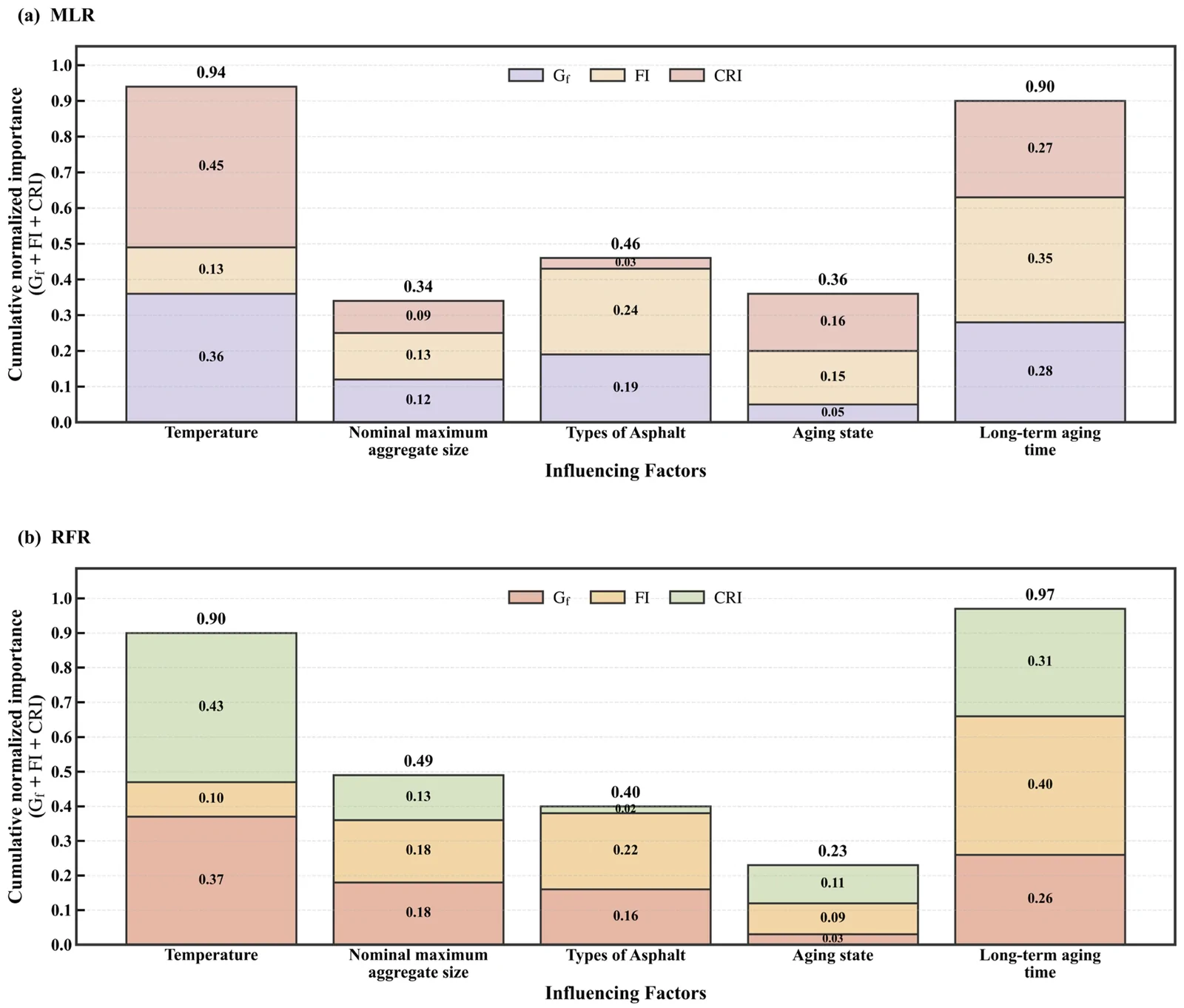

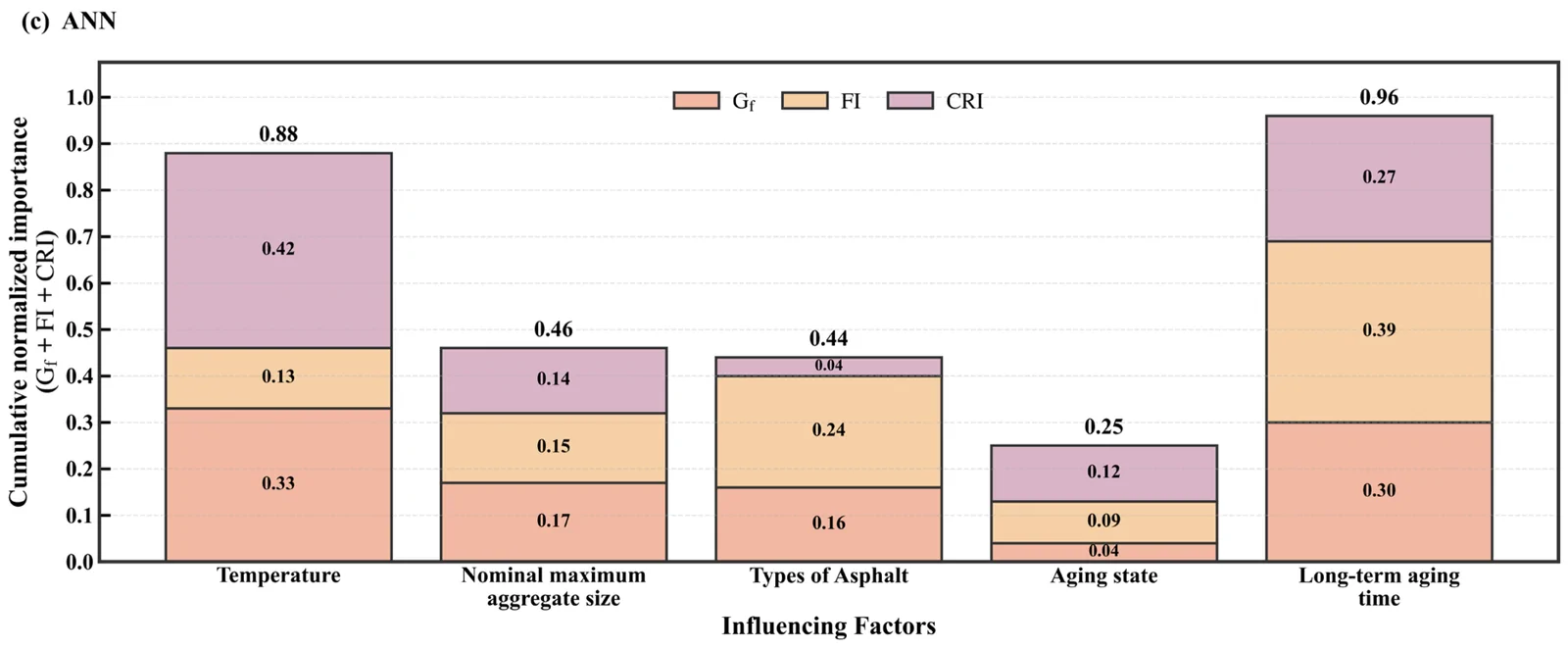
Ranking of Factors Across Models.

Figure 18 presents the normalized rankings of factor contributions obtained from the three model families. For *G_f_*, all three models consistently identify ambient temperature and long-term aging duration as the two most influential factors, indicating high sensitivity of *G_f_* to environmental exposure and service aging. This pattern suggests that energy accumulation and release prior to crack initiation are governed largely by stiffness evolution and aging-induced hardening. A closer comparison shows that MLR gives similar importance to temperature and long term aging, while RFR and ANN place slightly more weight on temperature, suggesting that a purely linear specification may under-represent temperature-driven variability. For FI, long term aging duration is the dominant contributor in all models, and binder type has higher relative importance for FI than for *G_f_* and *CRI*, indicating that *FI* reflects ductility and energy dissipation during crack propagation and is more responsive to viscoelastic toughness changes introduced by modified binders. For the *CRI*, ambient temperature exhibits an almost dominant contribution in all three models, followed by long-term aging, whereas the remaining factors show markedly lower importance, suggesting that *CRI* primarily reflects the overall cracking-risk response under thermal effects and is comparatively less sensitive to other mixture-related factors.

The MLR weights are relatively smooth under linear assumptions and may not fully capture the nonlinear patterns and interactions implied by qualitative observations. RFR enlarges the separation between primary and secondary factors for certain indices, consistent with the ability of tree-based models to capture higher-order interactions. ANN yields an intermediate pattern overall, while showing clearer discrimination between aging- and binder-related effects for *FI* and *CRI*, indicating an advantage in representing nonlinear viscoelastic responses.

The normalized importance scores across the different cracking indices were equally weighted and summed to obtain an integrated influence score. Across MLR, RFR, and ANN, ambient temperature and long-term aging duration consistently rank among the most important factors, confirming their dominant roles in governing the cracking-resistance evolution of ultra-thin overlays. In contrast, secondary factors vary across models: MLR tends to classify the short-term aging pretreatment condition and binder type as secondary contributors, whereas RFR and ANN more consistently place NMAS and binder type in the secondary tier. Considering model applicability and interpretability consistency, the ANN-based ranking is adopted as the overall conclusion: ambient temperature and long-term aging duration are primary factors; NMAS and binder type are secondary; and short-term aging pretreatment shows the lowest contribution to cracking resistance.

### 5.5. Independent Experimental Validation of Model Generalization

To further validate the predictive capability of the three models, an additional set of comparative experiments was conducted independent of the modeling dataset. These data were used as an external validation set to examine model generalization. The factor design of the comparative experiments is summarized in Table 12, and the corresponding prediction performance is reported in Table 13. The results show that the ANN achieves the lowest error metrics on the finite external validation set, outperforming both RFR and MLR in prediction accuracy. Accordingly, ANN is more suitable for predictive analysis of the cracking resistance of ultra-thin overlay asphalt mixtures and can provide more reliable technical support for performance estimation and engineering selection across different service conditions and mix design scenarios.

The results indicate that increasing binder viscosity and elasticity can improve mixture cohesion and delay crack initiation and propagation, thereby enhancing cracking resistance. However, excessive viscosity may reduce workability and affect aggregate coating and compaction quality, which may influence construction quality and long-term durability. Ultra-thin overlay mixture design should not focus on a single performance indicator, but should balance cracking resistance, high-temperature stability, construction workability, and long-term durability.

The present study still has some limitations. Fiber optimization was not included in the experimental design, and the effects of fiber type, content, length, and distribution on mixture performance were not evaluated. In ultra-thin overlay mixtures, fibers may improve asphalt mortar stability, enhance binder-aggregate interaction, and reduce raveling risk under traffic loading. They may also bridge microcracks and improve post-cracking resistance. Future studies should examine the interaction between fiber optimization and high-viscosity modified binders. Further material design should focus on modified binder technologies and fiber-reinforced systems with improved temperature stability and anti-aging capacity, aiming to achieve a more reliable balance among crack resistance, rutting resistance, and durability.

## 6. Conclusions

This paper systematically investigates the key factors governing the cracking resistance of asphalt mixtures used in asphalt ultra-thin overlays, including temperature, nominal maximum aggregate size, asphalt binder, short-term aging, and long-term aging. Three modeling approaches—multiple linear regression, random forest regression and artificial neural network regression—were applied and compared in terms of interpretability and predictive performance. The relative contributions of the influencing factors to fracture energy, flexibility index, and cracking resistance index were then quantified, ranked, and comprehensively assessed.

(1) Fracture energy and the flexibility index reached their highest values at low temperature and decreased as temperature increased, whereas the cracking resistance index showed an increasing trend with temperature. This opposite trend suggests that, within the investigated temperature range and mixture set, *CRI* may overemphasize post-peak ductility relative to peak-load degradation. Therefore, *CRI* may not fully reflect the strength degradation of asphalt mixtures in the intermediate-temperature range and should be interpreted together with *G_f_* and FI to provide a more comprehensive evaluation of cracking resistance.
(2) The high-viscosity and high-elasticity composite modified asphalt exhibits consistent cracking-response trends across mixtures with different nominal maximum aggregate sizes. Taking the representative SMA-8 mixture as an example, the modifier provides only limited improvement in fracture energy under low-temperature conditions, but shows superior stability as aging duration increases. Considering the changes in the flexibility index and the cracking index, its primary benefit is associated with enhanced ductility, improved stress relaxation, and increased energy dissipation in the crack-tip region, which strengthens post-peak toughening and delays crack propagation, rather than substantially increasing the load-carrying capacity at crack initiation.
(3) SMA 8 shows the best performance across all cracking resistance indices, indicating that an appropriate nominal maximum aggregate size improves the cracking resistance of asphalt mixtures. Very small or very large NMAS, such as SMA 5 or SMA 10, are associated with poorer performance. Fracture energy is more affected by temperature when evaluating aggregate size effects, and temperature variation can obscure the influence of NMAS.
(4) The aging process shows a clear three stage evolution. In the first stage (short term aging), *G_f_* increases relative to the unaged condition, and the increase is more evident at higher short term aging temperatures, while *FI* and *CRI* generally decrease. In the second stage (early long-term aging, 3 d), the short-term aging-related benefit in *G_f_* largely disappears and *G_f_* drops sharply, with FI and *CRI* continuing to decline, indicating pronounced deterioration. In the third stage (late long-term aging, 3–7 d), all indices still decrease but the reduction in *G_f_* becomes less steep; for *FI* and *CRI*, higher short term aging temperatures intensify the degradation throughout the aging process.
(5) The comparison among multiple linear regression, random forest regression, and artificial neural network models suggests that the cracking performance of asphalt mixtures used in ultra-thin overlays is affected by nonlinear relationships and multi-factor interactions. Accordingly, both the random forest and artificial neural network models achieve superior prediction accuracy and more stable generalization compared to the linear model, making them more suitable for performance evaluation and mechanism-oriented analysis, particularly under complex conditions.
(6) SHAP-based feature-effect interpretation was used to analyze the random forest and artificial neural network models, providing a data-driven description of the direction and nonlinear trends of factor effects. Compared with these models, multiple linear regression showed limitations in capturing the non-monotonic response of fracture energy to nominal maximum aggregate size, which may result in biased interpretation of the trend. Based on the ANN model interpretation, ambient temperature and long-term aging duration were identified as the most influential factors, followed by nominal maximum aggregate size and asphalt type, while short-term aging status showed a relatively limited contribution. In addition, the ANN showed the best performance on the limited external validation set used in this study, suggesting its potential applicability for predicting the performance of asphalt mixtures used in ultra-thin overlays within the investigated dataset.

## Figures and Tables

**Table 1:** Fundamental Properties of Mineral Materials.

Material	Size Fraction (mm)	Property	Test Result
Coarse aggregate	5–10	Apparent specific gravity (g/cm^3^)	2.890
	5–8		2.948
	3–5		2.933
	5–10	Water absorption (%)	1.75
	5–8		1.24
	3–5		1.37
	5–10	Los Angeles abrasion loss (%)	9.5
	5–8		10.3
	3–5		10.1
	5–10	Aggregate crushing value (%)	11.4
	5–8		10.9
	3–5		10.8
Fine aggregate	0–3	Apparent specific gravity (g/cm^3^)	2.944
		Soundness loss (%)	3.2
		Fine aggregate angularity (FAA) (%)	42
Mineral filler	-	Apparent specific gravity (g/cm^3^)	2.700

**Table 2:** Lignin fiber test specifications and technical requirements.

Index	Unit	Test Result
Fiber length	mm	0.9
Fiber diameter	μm	23
Oil absorption ratio	fold	6.1

**Table 3:** HVE modified additive test specifications and technical requirements.

Index	Unit	Test Result
Single-particle mass	g	0.017
Density	g/cm	0.981
Melt flow index	g/10 min	3.7
Ash content	%	0.38

**Table 4:** Test specifications and technical requirements for HVE composite modified asphalt.

Index	Unit	Test Result
Penetration (25 °C, 5 s, 100 g)	0.1 mm	43
Ductility (5 °C, 5 cm/min)	cm	38
Softening point (Ring-and-Ball, TR&B)	°C	101
Dynamic viscosity (60 °C)	Pa·s	>580,000
Brookfield rotational viscosity (170 °C)	Pa·s	2.243
Elastic Recovery (25 °C)	%	96

**Table 5:** Marshall volumetric properties of asphalt mixtures.

Aggregate Gradation Type	VV/%	VFA/%	VV/%
SMA-5	3.9	19.9	78.2
SMA-8	3.8	17.8	78.7
SMA-10	4.0	17.3	76.9

**Table 6:** Trial protocol grouping.

Index	Scope
Temperature (°C)	15, 25, 35
NMAS (mm)	5, 8, 10
Asphalt type	HVE composite modified asphalt, SBS-modified asphalt
Aged state	Unaged, 135 °C short-term aging, 175 °C short-term aging
Long-term aging time (d)	0, 3, 5, 7

**Table 7:** Variability statistics of replicate SCB test results.

Evaluation Index		Temperature (°C)	NMAS (mm)	Asphalt Type	Aged State	Long-Term Aging Time (d)
*G_f_*	mean range (95% CI)	2.8 to 4.2	3.3 to 3.8	3.4 to 3.7	3.1 to 3.8	3.2 to 4.1
	F-values	170.92	16.38	5.29	4.89	23.72
	*p*-values	<0.001	<0.001	0.022	0.037	<0.001
*FI*	mean range (95% CI)	9.1 to 13.2	8.6 to 12.5	9.2 to 11.7	9.3 to 20.4	4.8 to 17.6
	F-values	11.32	10.97	5.66	62.27	163.88
	*p*-values	<0.001	<0.001	0.018	<0.001	<0.001
*CRI*	mean range (95% CI)	567.9 to 992.5	687.6 to 843.1	758.5 to 868.4	674.9 to 1020.1	599.5 to 928.1
	F-values	167.75	11.78	8.42	36.65	38.25
	*p*-values	<0.001	<0.001	<0.001	<0.001	<0.001

**Table 8:** Optimal parameters for RFR.

Evaluation Index	Optimal Parameters			
	Number of Trees	Maximum Tree Depth	Minimum Samples Required to Split an Internal Node	Minimum Samples Required at a Leaf Node
*G_f_*	200	10	2	1
*FI*	500	15	2	1
*CRI*	500	15	2	1
Optimization Scope	(100, 200, 500)	(5, 10, 15)	(2, 5, 10)	(1, 2, 4)

**Table 9:** Optimal parameters for ANN.

Evaluation Index	Optimal Parameters				
	Number of Hidden Layers	Neurons per Hidden Layer	Activation Function	Learning Rate	L2 Regularization
*G_f_*	3	128	tanh	0.001	0.01
*FI*	1	128	relu	0.001	0.001
*CRI*	3	128	relu	0.001	0.01
Optimization Scope	(1, 2, 3)	(32, 64, 128)	(tanh, relu)	(0.0005, 0.001)	(0.01, 0.001, 0.0001)

**Table 10:** Fitting results for each model.

Model	Evaluation Index	R^2^	RMSE	MAE
MLR	*G_f_*	0.819	0.416	0.336
	*FI*	0.923	2.653	2.027
	*CRI*	0.921	65.230	53.117
RFR	*G_f_*	0.911	0.242	0.193
	*FI*	0.956	1.341	0.986
	*CRI*	0.950	48.846	40.542
ANN	*G_f_*	0.976	0.126	0.094
	*FI*	0.972	1.075	0.796
	*CRI*	0.961	43.104	34.887

**Table 11:** Multiple linear regression results for cracking performance indices.

Evaluation Index	Factor	Standardized Beta	*t*-Value	*p*-Value
*G_f_*	Temperature	−0.62	−25.926	<0.01
	NMAS	0.2	11.685	<0.01
	Asphalt type	0.32	12.374	<0.01
	Aged state	0.08	2.911	0.04
	Long-term aging time	−0.49	−19.335	<0.01
*FI*	Temperature	−0.25	−12.778	<0.01
	NMAS	0.25	12.788	<0.01
	Asphalt type	0.44	21.02	<0.01
	Aged state	−0.29	−12.821	<0.01
	Long-term aging time	−0.67	−32.341	<0.01
*CRI*	Temperature	0.75	49.512	<0.01
	NMAS	0.15	9.855	<0.01
	Asphalt type	−0.05	−2.911	0.04
	Aged state	−0.26	−14.845	<0.01
	Long-term aging time	−0.45	−28.044	<0.01

**Table 12:** Comparative Test Factor Design.

Evaluation Index	Asphalt Type	NMAS (mm)	Test Temperature (°C)	Aged State	Long-Term Aging Time (d)
*G_f_*	20	5	HVE composite modified asphalt	0	0
	20	8	SBS-modified asphalt	135 °C short-term aging	2
*FI*	20	10	HVE composite modified asphalt	175 °C short-term aging	4
	30	5	SBS-modified asphalt	135 °C short-term aging	6
*CRI*	30	8	SBS-modified asphalt	135 °C short-term aging	0
	30	10	HVE composite modified asphalt	175 °C short-term aging	2

**Table 13:** Model Comparison Results.

Actual Measured	Model					
	MLR		RFR		ANN	
	Predicted	Error	Predicted	Error	Predicted	Error
3.74	3.8	1.60%	3.78	1.07%	3.76	0.53%
4.32	3.73	−13.66%	4.21	−2.55%	4.41	2.08%
11.84	7.79	−34.21%	14.4	21.62%	10.02	−15.37%
2.8	3.5	25.00%	3.18	13.57%	2.98	6.43%
970.6	1085.04	11.79%	920.88	−5.12%	1006.65	3.71%
810.6	1106.22	36.47%	742.4	−8.41%	835.2	3.03%

## Data Availability

The original contributions presented in this study are included in the article. Further inquiries can be directed to the corresponding authors.

## References

[1] Cui W., Wu K., Cai X., Tang H., Huang W. (2020). Optimizing gradation design for ultra-thin wearing course asphalt. Materials.

[2] Traseira-Piñeiro L., Parry T., Haughey F., Garcia-Hernandez A. (2022). Performance of plant-produced asphalt containing cellular capsules. Materials.

[3] Shen C., Wu Z., Xiao P., Kang A., Wang Y. (2024). Experimental research on the anti-reflection crack performance of basalt fiber modified rubber asphalt stress-absorbing layer. Materials.

[4] Xiao M., Chen Y., Feng H., Huang T., Xiong K., Zhu Y. (2024). Evaluation of fatigue behavior of asphalt field cores using discrete element modeling. Materials.

[5] Wu X., Kang A., Wu B., Lou K., Fan Z. (2021). Prediction of crack resistance of LFSMA-13 with and without anti-rut agent using parameters of FTIR spectrum under different aging degrees. Materials.

[6] Yan Y., Zhang H., Bekoe M., Allen C., Zhou J., Roque R.J.C. (2023). Effects of asphalt binder type, aggregate type, and gradation characteristics on fracture properties and performance of asphalt mixtures at intermediate temperatures. Constr. Build. Mater..

[7] Ge S., Yuan J., Xiang Q., Xiao F. (2026). Ultrathin wearing course for pavement preventive maintenance: A state-of-the-art review. J. Transp. Eng. Part B Pavements.

[8] Ji X., Yuan Y., Huang Y., Shao J., Li S. (2024). Performance study of asphalt mixtures reinforced with gradated basalt fibers of mixed lengths. Materials.

[9] Yang S., Zhou Z., Li K. (2023). Experimental study on the cracking resistance of asphalt mixture with different degrees of aging. Appl. Sci..

[10] Liu S., Zhang Z., Wang R. (2023). Performance evaluation of asphalt concrete incorporating steel slag powder as filler under the combined damage of temperature and moisture. Sustainability.

[11] Wang J., Sun J., Luo S., Li Q. (2022). Laboratory and field performance evaluation of high-workability ultra-thin asphalt overlays. Materials.

[12] Zieliński P., Klimczak M., Tekieli M., Strzępek M. (2025). An evaluation of the fracture properties of asphalt concrete mixes using the semi-circular bending method and digital image correlation. Materials.

[13] Safazadeh F., Romero P., Mohammad Asib A.S., VanFrank K. (2022). Methods to evaluate intermediate temperature properties of asphalt mixtures by the semi-circular bending (SCB) test. Road Mater. Pavement Des..

[14] Ishaq M.A., Giustozzi F. (2021). Correlation between rheological fatigue tests on bitumen and various cracking tests on asphalt mixtures. Materials.

[15] Xia Y., Lin J., Chen Z., Cai J., Hong J., Zhu X. (2022). Fatigue Cracking Evolution and Model of Cold Recycled Asphalt Mixtures during Different Curing Times. Materials.

[16] Zhang Y., Zhang Y., Li B., Kang A., Wang Y. (2024). Evaluation of Cracking Resistance of SMA-13 Hot Recycling Asphalt Mixtures Reinforced by Basalt Fiber. Materials.

[17] Al-Qadi I.L., Ozer H., Zhu Z., Singhvi P., Mohamed Ali U., Sawalha M., Zehr T.G. (2019). Development of Long-Term Aging Protocol for Implementation of the Illinois Flexibility Index Test (I-FIT).

[18] Liu J., Liu F., Zheng C., Zhou D., Wang L. (2022). Optimizing asphalt mix design through predicting effective asphalt content and absorbed asphalt content using machine learning. Constr. Build. Mater..

[19] Khiavi A.J., Naeim B., Soleimanzadeh M. (2025). Development of a novel ensemble learning model for predicting asphalt volumetric properties using experimental data for pavement performance assessment. Sci. Rep..

[20] Kim D.-H., Kim H.-Y., Moon K.-H., Jeong J.-H. (2022). Prediction of Fracture Toughness of Intermediate Layer of Asphalt Pavements Using Artificial Neural Network. Sustainability.

[21] Nguyen L.N., Le T.-H., Nguyen L.Q., Tran V.Q. (2023). Machine learning approaches for predicting Cracking Tolerance Index (CTIndex) of asphalt concrete containing reclaimed asphalt pavement. PLoS ONE.

[22] Wu Z., Li S., Wang D., Qiu M., Fang C., Yang J., Tang H. (2025). Machine learning prediction of road performance of cold recycled mix asphalt with genetic algorithm hyperparameter optimization. Materials.

[23] (2014). Preparing and Determining the Density of Asphalt Mixture Specimens by Means of the Superpave Gyratory Compactor.

[24] American Association of State Highway and Transportation Officials (AASHTO) (2024). Short-Term Laboratory Conditioning of Asphalt Mixtures.

[25] American Association of State Highway and Transportation Officials (AASHTO) (2024). Long-Term Laboratory Conditioning of Asphalt Mixtures.

[26] Al-Qadi I.L., Ozer H., Lambros J., El Khatib A., Singhvi P., Khan T., Rivera-Perez J., Doll B. (2015). A Testing Framework for Evaluating Asphalt Mixture Performance Incorporating RAP and RAS.

[27] Ozer H., Al-Qadi I.L., Lambros J., El-Khatib A., Singhvi P., Doll B. (2016). Development of the fracture-based flexibility index for asphalt concrete cracking potential using modified semi-circle bending test parameters. Constr. Build. Mater..

[28] Cai Y., Sun H., Tao Z., Fu K., Yin H., Chen M., Zhuang S., Ren J. (2026). SMA-13 Recycled Asphalt Mixtures with Flocculent Basalt Fiber: Experiment and Random Forest Analysis. Materials.

[29] Geurts P., Ernst D., Wehenkel L. (2006). Extremely randomized trees. Mach. Learn..

[30] Abiodun O.I., Jantan A., Omolara A.E., Dada K.V., Mohamed N.A., Arshad H. (2018). State-of-the-art in artificial neural network applications: A survey. Heliyon.

[31] Goyal M., Goyal R., Lall B. (2019). Learning activation functions: A new paradigm for understanding neural networks. arXiv.

[32] El-Badawy S., Abd El-Hakim R., Awed A. (2018). Comparing artificial neural networks with regression models for hot-mix asphalt dynamic modulus prediction. J. Mater. Civ. Eng..

